# The ZEB1 pathway links glioblastoma initiation, invasion and chemoresistance

**DOI:** 10.1002/emmm.201302827

**Published:** 2013-07-01

**Authors:** Florian A Siebzehnrubl, Daniel J Silver, Bugra Tugertimur, Loic P Deleyrolle, Dorit Siebzehnrubl, Matthew R Sarkisian, Kelly G Devers, Antony T Yachnis, Marius D Kupper, Daniel Neal, Nancy H Nabilsi, Michael P Kladde, Oleg Suslov, Simone Brabletz, Thomas Brabletz, Brent A Reynolds, Dennis A Steindler

**Affiliations:** 1 Department of Neurosurgery, University of FloridaGainesville, FL, USA; 2 Department of Neuroscience, University of FloridaGainesville, FL, USA; 3 Department of Pathology, University of FloridaGainesville, FL, USA; 4 Institute for Reconstructive Neurobiology, University of BonnGermany; 5 Department of Biochemistry and Molecular Biology, University of Florida Shands Cancer Center Program in Cancer Genetics, Epigenetics and Tumor VirologyGainesville, FL, USA; 6 Department of Visceral Surgery, University Hospital FreiburgFreiburg, Germany

**Keywords:** brain, cancer stem cell, EMT, glioma, xenograft

## Abstract

Glioblastoma remains one of the most lethal types of cancer, and is the most common brain tumour in adults. In particular, tumour recurrence after surgical resection and radiation invariably occurs regardless of aggressive chemotherapy. Here, we provide evidence that the transcription factor ZEB1 (zinc finger E-box binding homeobox 1) exerts simultaneous influence over invasion, chemoresistance and tumourigenesis in glioblastoma. ZEB1 is preferentially expressed in invasive glioblastoma cells, where the ZEB1-miR-200 feedback loop interconnects these processes through the downstream effectors ROBO1, c-MYB and MGMT. Moreover, ZEB1 expression in glioblastoma patients is predictive of shorter survival and poor Temozolomide response. Our findings indicate that this regulator of epithelial-mesenchymal transition orchestrates key features of cancer stem cells in malignant glioma and identify ROBO1, OLIG2, CD133 and MGMT as novel targets of the ZEB1 pathway. Thus, ZEB1 is an important candidate molecule for glioblastoma recurrence, a marker of invasive tumour cells and a potential therapeutic target, along with its downstream effectors.

Glioblastoma have a poor prognosis, mainly due to infiltrating and therapy resistant cells leading to cancer recurrence. Here, tumor formation, invasion and resistance are not independent but intertwined processes regulated by the EMT activator ZEB1.

## INTRODUCTION

With a median survival of about 15 months (Schwartzbaum et al, 2006; Stupp et al, 2005), glioblastoma is the most frequent and aggressive of all gliomas, with a propensity to invade the surrounding parenchyma (Wen & Kesari, 2008). Individual tumour cells can be found far from the primary tumour site, often crossing great distances into the contralateral hemisphere (Wilson, 1992). These cells cannot be isolated for surgical resection, or easily targeted by irradiation, and thus represent sources for tumour recurrences (Glas et al, 2010). Adjuvant chemotherapy (*e.g*. Temozolomide, TMZ) is therefore included as a critical component of the current standard of care, in attempt to address these residual invasive cells. Given the exceedingly poor prognosis, it is critical to understand the biology of treatment-resistant glioblastoma cells.

The cancer stem cell hypothesis (Reya et al, 2001) postulates an intra-tumoural hierarchy, where a small population of tumour cells has greater abilities to initiate and propagate tumours (Ignatova et al, 2002), rendering cancer stem cells an important therapeutic target (Vescovi et al, 2006). Cancer stem cells have been shown to be more invasive and therapy resistant than other cells of the same tumours (Bao et al, 2006; Cheng et al, 2011; Lathia et al, 2011). Tumour heterogeneity is a direct implication of the cancer stem cell hypothesis, and indicates that cell populations with different properties (such as drug resistance or higher capacity for tumour/recurrence formation) exist within the same tumour (Siebzehnrubl et al, 2011). The triad of tumourigenesis (cancer stemness), invasion and therapy resistance is a fatal combination if merged in a single cell population, and renders such a population an important contributor to poor outcome.

This triad is induced by Epithelial–Mesenchymal Transition (EMT) in cancers outside the CNS, where EMT is the major cause of invasion and metastasis (Chaffer & Weinberg, 2011), and cancer cells undergoing EMT have been shown to acquire stem cell traits and are frequently more therapy resistant (Mani et al, 2008; Polyak & Weinberg, 2009; Singh & Settleman, 2010). Therefore, EMT can generate cell populations that combine these three above-mentioned hallmarks. However, the role of EMT and related processes in brain cancer has received little attention thus far (Kahlert et al, 2012; Lu et al, 2012; Mikheeva et al, 2010), likely because the brain is lacking critical tissue components (*i.e*. epithelium and mesenchyme). Yet, it is conceivable that key invasion pathways overlap between CNS and other cancers, and that factors inducing EMT outside the brain also activate the triad of invasion, stemness and chemoresistance in malignant gliomas.

Many factors, including reduced cell adhesion (Asano et al, 2004), reduced matrix adhesion (Nakada et al, 2007), matrix protease secretion (Rao, 2003) and cytoskeletal remodeling (Giese et al, 1996) have been advanced as determinants of glioma invasion; several of these pathways are induced by EMT outside the CNS (Chaffer & Weinberg, 2011). Therapy resistance in glioma is mediated by expression of DNA repair enzymes (Bao et al, 2006), and/or expression of drug efflux transporters (Bleau et al, 2009). Of particular note, *O*-6-Methylguanine DNA Methyltransferase (MGMT) confers resistance to the standard of care drug TMZ (Bocangel et al, 2002). While MGMT is also expressed in several non-CNS cancers (Gerson, 2004), it is currently unknown whether EMT can induce MGMT expression. Glioma stemness has been linked to a number of transcription factors, such as SOX2 (Gangemi et al, 2009), OLIG2 (Ligon et al, 2007), and BMI1 (Facchino et al, 2010). SOX2 and BMI1 are targets of EMT activators, in particular of ZEB1 (Wellner et al, 2009).

ZEB1 is an inducer of EMT, transcriptional repressor of cell-adhesion molecules, miRNAs—particularly the miR-200 family—and cell polarity-associated genes (Brabletz & Brabletz, 2010; Wellner et al, 2009). It has emerged as one of the master regulators for metastasis (Brabletz & Brabletz, 2010) and plays a critical role in tumour initiation at distant sites (Wellner et al, 2009). Edwards et al (2011) showed induction of ZEB1 through the tumour microenvironment in glioma, and related ZEB1 expression to repression of E-cadherin and thus invasion. Given the low prevalence of E-cadherin in glioma (Utsuki et al, 2002), we asked whether ZEB1 could affect glioma invasion through other mechanisms. We further aimed to elucidate if ZEB1 regulates other, typically EMT-related processes in brain cancer, such as potential for recurrence and therapy resistance. Expression of ZEB1 has been observed in chemoresistant cells in cancers outside the CNS (Li et al, 2012; Wang et al, 2009), but whether this relation is correlative or causative is thus far unknown.

Our study provides a systematic analysis of the functions of ZEB1 in glioblastoma pathobiology. Specifically, we address how ZEB1 exerts its effects on key malignant processes in glioma, *i.e*. invasion, tumourigenesis and therapy resistance.

## RESULTS

### ZEB1 is expressed in invasive glioblastoma cells

**Figure 1:**
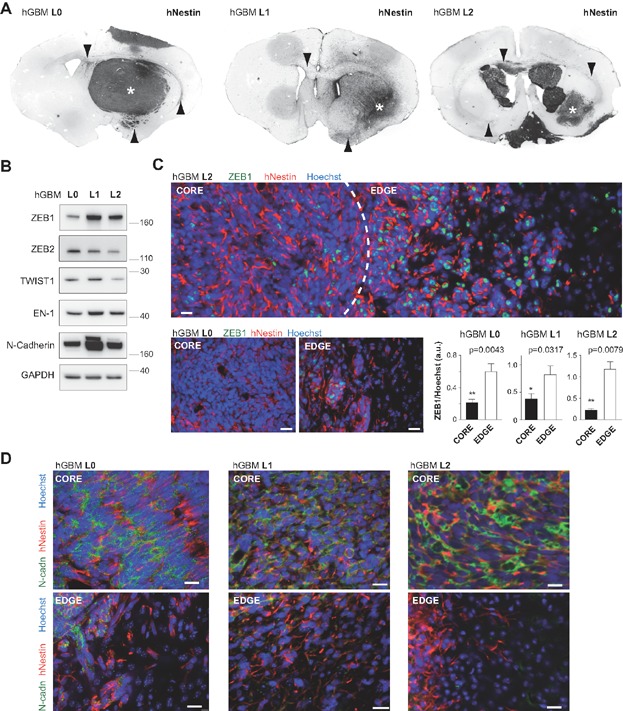
**ZEB1 is expressed in invasive tumour cells**Source data is available for this figure in the Supporting Information.Primary glioblastoma (hGBM) cell lines L0, L1 and L2 show varying degrees of tumour mass (asterisks) and invasion (arrowheads). Images are inverted black and white micrographs of fluorescent immunostainings for human-specific nestin (*n* = 5 animals each).EMT factors ZEB1, ZEB2, Twist1 and En-1, as well as N-cadherin are expressed in invasive hGBM cell lines. Additional markers are shown in Supporting Information Fig 1A.ZEB1 is highly expressed at the invasion front of tumours, but not within the tumuor mass (dotted line separates areas of tumour mass and invasion; single image projection from confocal *z*-stack, scale bars 20 µm). Bar graphs depict ratios of mean fluorescence intensity at randomly selected tumour core and edge areas (*n* = 5 each, Mann–Whitney *U*-test).While the tumour mass is immunopositive for N-cadherin, invasive cells are negative. Scale bars 20 µm.

Using three cell lines generated from primary glioblastoma specimens (hGBM L0, L1 and L2 (Deleyrolle et al, 2011; Piccirillo et al, 2006)), we observed varying degrees of tumour invasion in xenograft models (Fig 1A). In all cases, invasion followed the brain architecture, with tumour cells migrating along the subcortical white matter across the midline (Fig 1, arrowheads). We hypothesised that factors associated with EMT might also govern brain tumour invasion. Therefore, we studied the expression of EMT-associated factors in our cell lines, and found that ZEB1, ZEB2, Twist1 and Engrailed1, as well as N-cadherin are expressed across all three lines (Fig 1B). In contrast, Snail, Slug and E-cadherin were found in only one line (Supporting Information Fig S1A). Analysis of these factors in the TCGA dataset (The Cancer Genome Atlas Research Network, 2008) revealed that only ZEB1 showed a significant correlation with patient survival (Supporting Information Fig S1B). Hence, we chose to focus on this protein.

ZEB1 is preferentially localised at the invasion front in tumour xenografts (Fig 1C, Supporting Information Fig S1C). We observed that N-cadherin expression is confined to the tumour mass, tapering off towards the invasion front, and is absent in invasive cells (Fig 1D). Notably, an inverse relationship between N-cadherin expression and invasion has been described (Asano et al, 2004), and the importance of cell-cell interactions for EMT processes and plasticity has recently been discussed (Thompson & Haviv, 2011). Beta-catenin is known to induce expression of ZEB1 (Kahlert et al, 2012; Schmalhofer et al, 2009), but we did not observe nuclear accumulation of beta-catenin in our samples. Instead, immunoreactivity was confined to cell membranes within the tumour core, and absent at the invasion front, corroborating reduced cell–cell contacts during invasion (Supporting Information Fig S1D). Because the ZEB1-positive population was coincident with the distal-most, invading tumour cells, we hypothesised that ZEB1 may have a regulatory role in glioma invasion, and attempted to determine its specific functions in glioblastoma.

### ZEB1 knockdown reduces invasion and chemoresistance

**Figure 2:**
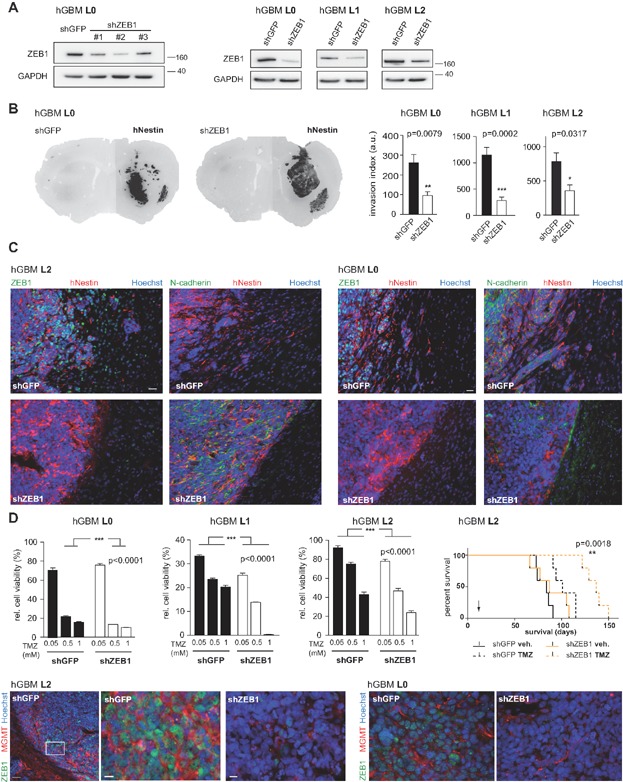
**ZEB1 knockdown reduces invasion and chemoresistance**Source data is available for this figure in the Supporting Information.Short hairpin constructs against ZEB1 prominently reduce its protein levels. shZEB1 targets ZEB1 protein in all three primary cell lines.ZEB1 knockdown results in reduced tumour invasion in all cell lines (*n* = 5 animals per group, Mann–Whitney *U*-test). Threshold images used to determine invasion index are shown in Supporting Information Fig S2C.Control tumours show expression of ZEB1, but not N-cadherin, at the invasion front, while tumours derived from ZEB1 knockdown cells are characterised by high levels of N-cadherin and no invasion. Scale bar 20 µm (applies to all panels).ZEB1 knockdown results in significantly greater sensitivity to TMZ in MTT cell viability assays (*n* = 8, one-way ANOVA) and in xenografted tumours (*n* = 5 animals each, log-rank test, arrow indicates TMZ treatment). Increased ZEB1 expression at the edges of control tumours is associated with expression of MGMT. ZEB1 knockdown tumours do not stain for MGMT. Scale bars 50 µm in left and 10 µm in other panels.

To address its functions in invasion we knocked down ZEB1 expression in glioblastoma cells. Three short hairpin RNA constructs (including one previously described (Wellner et al, 2009)) were used to knockdown ZEB1 in glioblastoma cells (Fig 2A). This resulted in increased localisation of N-cadherin and beta-catenin at the membrane (Supporting Information Fig S2A), as well as in higher proliferation (Supporting Information Fig S2B). Xenograft tumours of ZEB1 knockdown cells (shZEB1) were non-invasive, and consisted only of an expansive tumour mass (Fig 2B). Of note, invasion correlated with ZEB1 expression levels, and was highest in the L1 and lowest in the L0 primary line (Fig 2B, Fig 1A and B). Immunofluorescence demonstrated an inverse relationship between ZEB1 and N-cadherin at the invasion front of control tumours, while membrane-associated expression of N-cadherin was retained up to the sharply defined borders of the shZEB1-derived tumour mass (Fig 2C).

Since EMT has been associated with therapy resistance (Singh & Settleman, 2010), we next tested whether ZEB1 affects response of tumour cells to the standard of care drug TMZ. A cell viability analysis revealed that ZEB1 knockdown significantly increased TMZ sensitivity *in vitro* (Fig 2D). To validate whether ZEB1 promotes chemoresistance *in vivo*, we treated tumour-bearing animals grafted with either shZEB1 or shGFP cells with one cycle (five doses) of clinically relevant TMZ concentrations (20 mg/kg (Zhou et al, 2007)). This resulted in a significantly increased survival time of shZEB1 over shGFP animals (Fig 2D). Further, we observed that expression of MGMT, a major chemoresistance enzyme (Bocangel et al, 2002), increased towards the tumour edges in control tumours, and that ZEB1 and MGMT co-localise (Fig 2D). MGMT was localised to the cytoplasm, which has been described (Ishibashi et al, 1994).

Together, these data suggest that invasive cells are capable of evading chemotherapy and support a function of ZEB1 in both glioblastoma invasion and chemoresistance.

### Glioblastoma invasion is mediated through ZEB1 and ROBO

**Figure 3:**
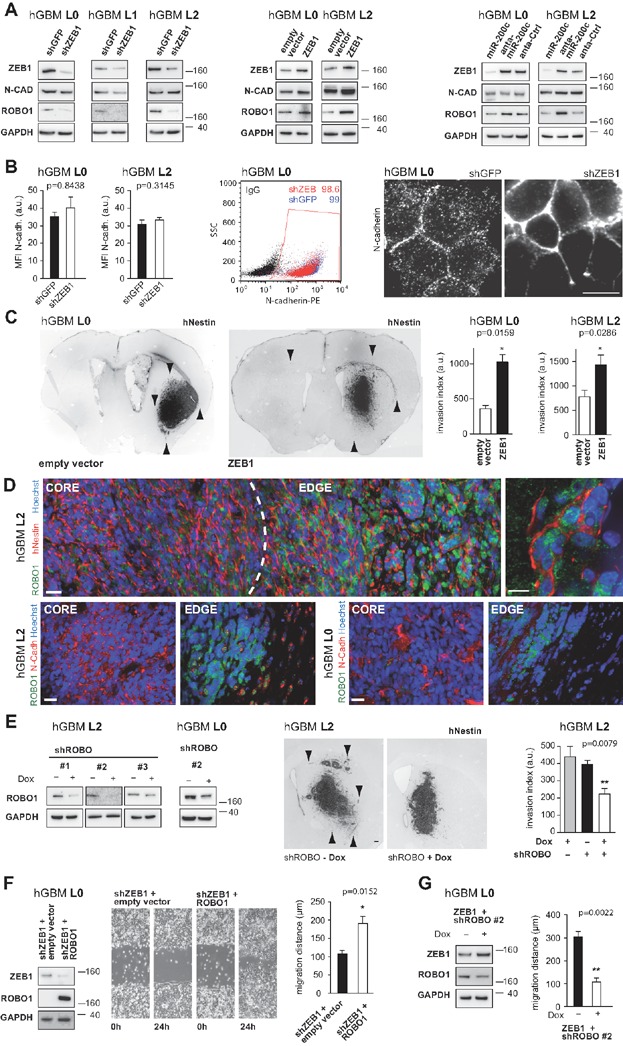
**ROBO is a downstream effector of ZEB1 that mediates invasion**Source data is available for this figure in the Supporting Information.Knockdown of ZEB1 has no effect on total protein levels of N-cadherin, but robustly depletes ROBO1. Forced expression of ZEB1 does not affect N-cadherin, but increases ROBO1 levels. Likewise, modulation of miR-200 expression affects expression of ROBO1.N-cadherin immunostaining detects similar levels on control and ZEB1 knockdown cells (MFI: mean fluorescence intensity; *n* = 11, Mann–Whitney *U*-test; additional images shown in Supporting Information Fig S3). Flow cytometry shows no difference in N-cadherin positive cell numbers in knockdown *versus* control (extracellular epitope; numbers reflect % positive cells). N-cadherin signal is speckled in control cells, but concentrated at cell–cell contact membranes in ZEB1 knockdown.Targeted expression of ZEB1 increases tumour invasion (*n* = 5 animals each, one-way ANOVA; arrowheads point to maximum invasion distances).ROBO1 is expressed at the tumour edge, and is inversely proportional to the expression of N-Cadherin. High magnification shows ROBO1-positive tumour cells distal to the invasion front. Scale bars, 10 µm.Inducible short hairpin constructs against ROBO1 can reduce its protein levels in the presence of doxycycline (Dox). shROBO xenografts show reduced invasion in the presence of Dox (*n* = 5 animals each; one-way ANOVA; arrowheads highlight extent of invasion in control conditions; additional data shown in Supporting Information Fig S3).ROBO1 rescues migration phenotype in ZEB1 knockdown cells (*n* = 3 for migration assay; additional data shown in Supporting Information Fig S3).ROBO1 knockdown depletes migration of ZEB1 overexpressing cells (*n* = 3, additional data shown in Supporting Information Fig S3).

Based on our observation of reduced membrane-associated N-cadherin in invasive glioblastoma cells, we speculated that reduced cell–cell contacts contribute to greater invasiveness of ZEB1-positive cells. Curiously, although immunofluorescent analysis of xenografted tumours indicated that invasive tumours lacked N-cadherin (Fig 2C), Western analysis revealed that overall N-cadherin protein levels did not change between ZEB1 knockdown and controls (Fig 3A). Corroborating our immunoblot observations, there was no significant difference in the mean fluorescence intensity of N-cadherin between ZEB1 knockdown and control cells *in vitro*. Flow cytometry analysis of live cells demonstrated that fluorescence intensity as well as the number of labelled cells was similar between ZEB1 knockdown and controls (Fig 3B). Since the antibody used was directed against the extracellular epitope of N-cadherin, this indicated that N-cadherin was not internalised in invasive cells.

We therefore hypothesised that the distribution of N-cadherin might provide the critical difference for tumour cell adhesion. Closer scrutiny of N-cadherin immunostaining confirmed that membrane distribution of this protein differed between control and ZEB1 knockdown cells. In control cells, N-cadherin protein stippled the cell membrane evenly, whereas ZEB1 knockdown resulted in the concentration of N-cadherin to the juxtaposed membranes between adjacent cells (Fig 3B). Re-distribution of N-cadherin from the area of contact between cells may account for greater cell motility and may be achieved if N-cadherin is disconnected from its intracellular anchor to the cytoskeleton. The axon guidance molecule ROBO1 can sever the anchorage of N-cadherin to the cytoskeleton (Rhee et al, 2002), thereby increasing cellular motility, and is expressed in malignant glioma (Mertsch et al, 2008).

A database search for microRNA binding sites revealed ROBO1 as a potential target of the ZEB1 regulatory loop (Ghosh, 2000; John et al, 2004). Therefore, we scrutinised ROBO1 as intermediary regulator of invasion. Consistently, we found that ZEB1 knockdown reduced expression levels of ROBO1, while overexpression of ZEB1 had the opposite effect (Fig 3A). Additionally, ROBO1 is located at the outer membrane of control, but not ZEB1 knockdown cells (Supporting Information Fig S3A). Overexpressing or antagonising miR-200c decreased or increased ROBO1 expression, respectively (Fig 3A). Similarly, these manipulations restored normal ROBO1 expression levels in ZEB1 overexpressing and knockdown cells (Supporting Information Fig S3B). Antagonising miR-200c also increased cell migration *in vitro* (Supporting Information Fig S3C).

Forced expression of ZEB1 resulted in greater invasiveness of xenograft tumours, with tumour cells covering large distances along white matter tracts (Fig 3C, arrowheads). Within tumours, ROBO1 expression increases towards the invasion front, and is inversely proportional to the expression of N-cadherin (Fig 3D). We next tested whether interference with ROBO1 has direct effects on tumour cell migration and invasion. Using three inducible shRNA constructs against its sequence, we observed a prominent, doxycycline-dependent reduction in ROBO1 protein expression in two cell lines (Fig 3E). In an *in vitro* scratch assay, doxycycline-induced knockdown of ROBO1 prominently inhibited cell migration (Supporting Information Fig S3D). Expression of ROBO could be rescued by a non-targeted construct (Supporting Information Fig S3E). Importantly, shROBO1 cells gave rise to less invasive tumours in animals that were treated with doxycycline (Fig 3E, Supporting Information Fig S3F). Finally, the migratory phenotype of ZEB1 knockdown cells could be rescued by overexpressing ROBO1 (Fig 3F, Supporting Information Fig S3G), while blocking ROBO1 prominently reduced migration of ZEB1 overexpressing cells (Fig 3G, Supporting Information Fig S3G). These data support the notion that ROBO1 is regulated by ZEB1, and that ROBO1 is likewise another potential candidate molecule for regulating glioblastoma invasion.

### ZEB1 regulates MGMT via miR-200c and c-MYB to promote chemoresistance

**Figure 4:**
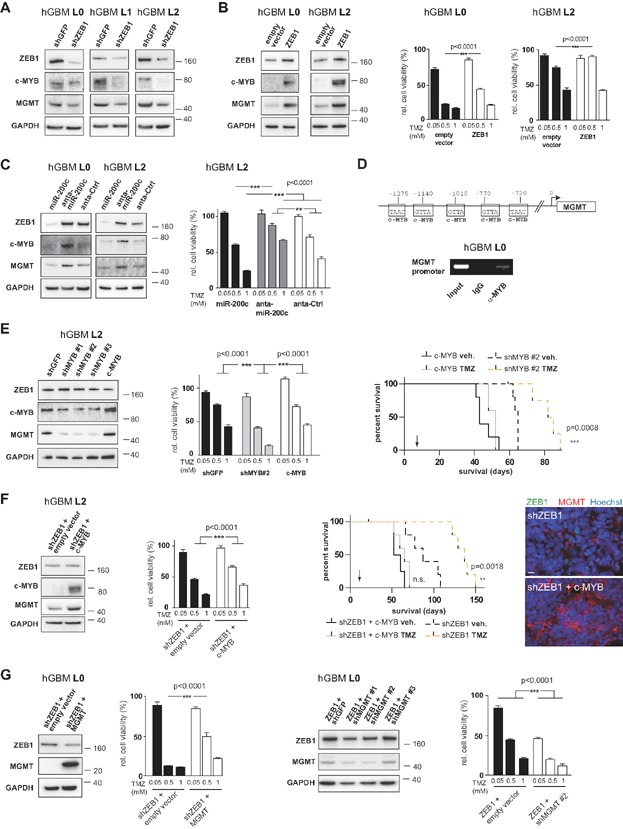
**ZEB1 regulates MGMT expression and chemoresistance through miR-200c and c-MYB**Source data is available for this figure in the Supporting Information.ZEB1 knockdown reduces protein levels of c-MYB and MGMT.Expression of ZEB1 increases protein levels of c-MYB and MGMT, and results in higher resistance to TMZ *in vitro*.miR-200c affects protein expression of ZEB1, c-MYB and MGMT, while antagonising miR-200c has opposite effects. Modulation of miR-200c affects chemoresistance to TMZ (*n* = 8, one-way ANOVA).Chromatin immunoprecipitation using anti-MYB antibody demonstrates c-MYB binding to the MGMT promoter in primary glioblastoma cells. Indicated are putative c-MYB binding sites in the *MGMT* promoter (Ghosh, 2000).Knockdown of c-MYB reduces expression of MGMT, while overexpression of c-MYB increases MGMT levels. Both have no influence on ZEB1 expression. Resistance to TMZ is significantly reduced by c-MYB knockdown *in vitro* (*n* = 8, one-way ANOVA). Ablation of c-MYB increases survival of tumour-bearing animals, while overexpression of c-MYB negates TMZ effects (*n* = 5 animals each, log rank test). Experiments with an additional cell line are shown in Supporting Information Fig 4.Chemoresistance in ZEB1-knockdown cells can be rescued by expression of c-MYB (*n* = 8, one-way ANOVA). Knockdown of ZEB1 and simultaneous expression of c-MYB abolish differences in survival times after TMZ treatment (*n* = 5 animals each, log-rank test). Tumours from ZEB1 knockdown, c-MYB expressing animals are immunoreactive for MGMT, but not ZEB1. Scale bar, 10 µm (applies to both images)Chemoresistance in ZEB1 knockdown cells can be rescued by expression of MGMT (*n* = 8, one-way ANOVA), and chemoresistance of ZEB1 expressing cells can be ablated by knockdown of MGMT (*n* = 8, one-way ANOVA).

We postulated above that EMT-associated factors might govern increased chemoresistance of invasive cells. Since ZEB1 knockdown cells are indeed more sensitive to TMZ (Fig 2D), we tried to resolve the underlying mechanism. We speculated that ZEB1 mediates chemoresistance of invasive cells through transcriptional regulation of MGMT, and confirmed reduced MGMT levels after ZEB1 knockdown in immunoblots (Fig 4A). MGMT is unlikely a direct target of the ZEB1 loop based on sequence analysis for binding sites of microRNAs (Ghosh, 2000; John et al, 2004). However, bioinformatics analysis of the *MGMT* promoter from position −1500 to +10 relative to the transcriptional start site (TSS; Ghosh, 2000) revealed five potential binding sites for the proto-oncogene c-MYB, which is regulated by miR-200 (Cesi et al, 2011). We found c-MYB depleted in ZEB1 knockdown cells (Fig 4A), indicating it as a potential intermediary between ZEB1/miR-200 and MGMT.

Targeted expression of ZEB1 induced c-MYB and MGMT (Fig 4B, Supporting Information Fig S4), and increased TMZ resistance *in vitro* (Fig 4B). Increasing or decreasing levels of miR-200c elicited similar changes in c-MYB and MGMT, as well as chemoresistance (Fig 4C). Antagonising miR-200c restored expression of c-MYB and MGMT as well as chemoresistance in ZEB1 knockdown cells (Supporting Information Fig S4A), while overexpressing miR-200c depleted expression of c-MYB and MGMT and reduced chemoresistance in ZEB1 overexpressing cells (Supporting Information Fig S4B). These findings substantiated that ZEB1 may mediate glioma chemoresistance through miR-200c and c-MYB.

To further characterise this pathway (ZEB1/miR-200/c-MYB), we performed additional experiments to test the role of miR-200c and c-MYB in chemoresistance. Using chromatin immunoprecipitation, we confirmed that c-MYB binds to the *MGMT* promoter in glioblastoma cells (Fig 4D). c-MYB knockdown reduced expression of MGMT, which was accompanied by reduced TMZ resistance *in vitro* and *in vivo* (Fig 4E, Supporting Information Fig S4C). In contrast, overexpression of c-MYB induced MGMT, and increased chemoresistance. Further, chemoresistance and MGMT expression of ZEB1-knockdown cells could be rescued by over-expressing c-MYB in these cells (Fig 4F, Supporting Information Fig S4D). MGMT was expressed independently of ZEB1 in these tumours, supporting c-MYB as intermediate regulator of MGMT. In contrast, knockdown of c-MYB reduced MGMT expression and chemoresistance of ZEB1 overexpressing cells (Supporting Information Fig S4E). Finally, chemoresistance of shZEB1 cells could be rescued by overexpression of MGMT, while knocking down MGMT in ZEB1-overexpressing cells increased their chemosensitivity (Fig 4G). Knockdown of ZEB1, c-MYB and MGMT could be rescued by non-targeted constructs (Supporting Information Fig S4F).

Differences in MGMT expression and chemoresistance have been attributed to methylation of the *MGMT* promoter (Hegi et al, 2005). To elucidate whether methylation differences affect MGMT expression in the context of ZEB1, we performed bisulfite genomic sequencing analysis on the region from −552 to +120 relative to the MGMT TSS, and found no significant differences in methylation between shGFP and shZEB1 knockdown cells in any of our cell lines (Supporting Information Fig S5). These findings demonstrate that ZEB1 induces chemoresistance through transcriptional regulation of MGMT via miR-200c and c-MYB.

### The ZEB1-miR-200 loop regulates stemness and tumour initiation

**Figure 5:**
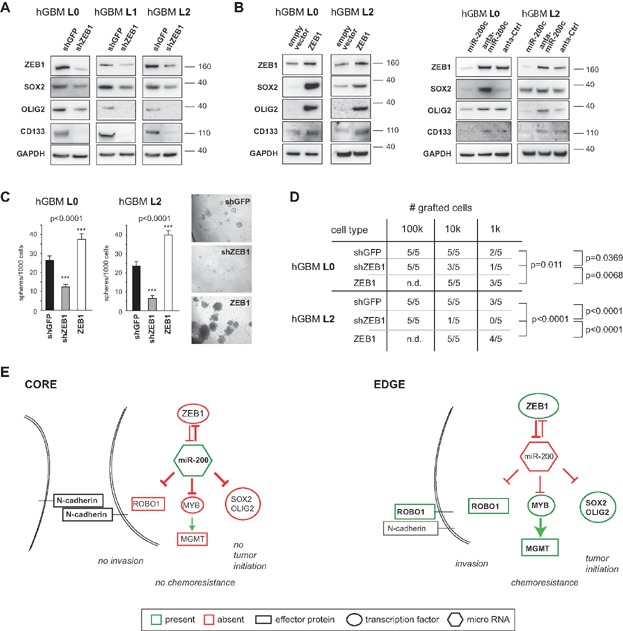
**ZEB1 regulates stemness and tumour formation**Source data is available for this figure in the Supporting Information.ZEB1 knockdown reduces protein levels of SOX2, OLIG2 and CD133.Expression of ZEB1 increases protein levels of these proteins. Expression of miR-200c reduces, and antagonising miR-200c increases SOX2, OLIG2 and CD133 levels.Tumour sphere formation is decreased by ZEB1 knockdown, and increased by its expression (*n* = 8, one-way ANOVA).ZEB1 knockdown significantly reduced intracerebral tumour formation, whereas expression of ZEB1 resulted in increased tumour formation frequency (chi square test).Schematic model depicts interconnection between invasion, chemoresistance and tumour initiation. Invasive cells express high levels of ZEB1, which inhibits the expression of miR-200. ROBO1 and c-MYB are consequently expressed and affect cell adhesion (by way of ROBO1 activity and the disconnection of N-cadherin from the cytoskeleton) and chemoresistance (by way of c-MYB and the induction of MGMT). SOX2 and OLIG2 mediate stemness and tumour initiation capacities in these cells. Non-invasive cells in the tumour mass do not express ZEB1; therefore microRNAs inhibit expression of SOX2, OLIG2, ROBO1 and c-MYB. N-cadherin remains tethered to the cytoskeleton and inhibits cell motility. MGMT is not expressed and cells are more sensitive to TMZ.

Since increased invasion (Cheng et al, 2011) and therapy resistance (Bao et al, 2006; Chen et al, 2012) have been observed in cancer stem cells (Venere et al, 2011), and ZEB1 is a known regulator of stemness and SOX2 in other solid tissue cancers (Wellner et al, 2009), we hypothesised that ZEB1 has similar functions in glioblastoma. We identified miR-200c binding sites in the sequences of the stemness-promoting transcription factors SOX2 and OLIG2, as well as the stem cell surface marker CD133/PROM1 (Supporting Information Fig S2) (John et al, 2004). Therefore, we tested whether the ZEB1-miR-200 feedback loop influenced expression of these proteins. Indeed, knockdown of ZEB1 decreased protein levels of SOX2, OLIG2 and CD133, while ZEB1 overexpression had opposite effects (Fig 5A and B). Overexpressing or antagonising miR-200c inversely affected expression of SOX2, OLIG2 and CD133, supporting inhibition of these factors by miR-200c. In a functional assay for stemness, decreasing or increasing ZEB1 expression resulted in lower or higher sphere-forming frequency, respectively (Fig 5C). Importantly, orthotopic grafts of shZEB1 cells showed a significantly decreased capacity for tumour initiation compared to control or ZEB1-expressing cells (Fig 5D). We conclude that ZEB1 is a regulator of stemness in glioblastoma.

In summary, we found that invasive cells can be distinguished from a xenografted brain tumour mass by their expression of ZEB1 (Fig 5E). Generally, tumour mass cells are stationary due to increased cell-cell adhesion, chemosensitive due to lower expression of MGMT, and less capable of tumour formation due to their lower stemness. In contrast, ROBO1 disconnects N-cadherin from the cytoskeleton in invasive cells, thus increasing their motility; c-MYB induces expression of MGMT, resulting in higher chemoresistance; expression of SOX2 and OLIG2 result in greater stemness and higher capacity for tumour formation. All these processes appear to be governed at least in part by ZEB1 and miR-200c.

### Protein levels of ZEB1 are predictive of clinical outcome

**Figure 6:**
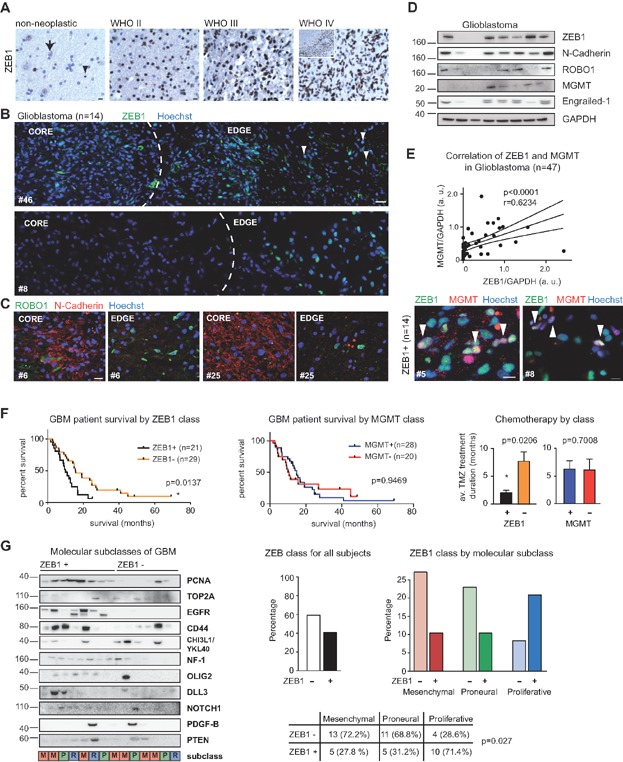
**ZEB1 expression is associated with unfavourable outcome in glioblastoma patients**Source data is available for this figure in the Supporting Information.ZEB1 is weakly reactive in some neurons (arrow) and strongly reactive in scattered glial cells (arrowhead) in non-neoplastic parenchyma, and labels glioma cells with increasing frequency and intensity with progressing malignancy (grade II astrocytoma, grade III anaplastic astrocytoma and grade IV glioblastoma; inset in grade IV depicts pseudopalisading necrosis; scale bars 10 µm).In glioblastoma, ZEB1 expression is highest at the invasive edge, which is mirrored by expression of ROBO1.N-cadherin is most prominent at the core, and virtually absent at the edge (representative images from 14 cases, scale bars 20 µm). Representative images for ZEB1 negative tumours are shown in Supporting Information Fig S5.EMT markers ZEB1 and En-1 are expressed in glioblastoma samples, as well as N-cadherin and MGMT. Additional immunoblots for EMT-associated proteins are shown in Supporting Information Fig S6.Expression of MGMT and ZEB1 are correlated (*n* = 47, Spearman Correlation). Within ZEB1-expressing tumours, MGMT is frequently co-expressed in ZEB1+ cells (arrowheads; 51 ± 9% co-localisation; *n* = 14; see also Supporting Information Fig S6). Nuclei are counterstained with Hoechst. Scale bar 10 µm.Immunoblotting analyses in 50 patient tumour specimens revealed that approx. 40–45% of glioblastomas express ZEB1. This is correlated with clinical outcome (median survival ZEB1+ 10.2 months, ZEB1− 15.7 months, log-rank test). While comparable specimen numbers expressed MGMT, no significant correlation with outcome was found. Response to TMZ treatment (average TMZ treatment duration ZEB1+ 2.0 months, ZEB1− 7.7 months, Mann–Whitney test) was also significantly extended for ZEB1+, but not for MGMT+ specimens.Stratification of glioblastoma specimens into molecular subclasses revealed a significantly higher portion of ZEB1+ specimens in the proliferative subclass, while more ZEB1− specimens were grouped into the mesenchymal and proneural subclasses. Analysis of individual markers across specimens is presented in Supporting Information Fig S6.

Having established a molecular model for the actions of ZEB1 in glioblastoma, we sought to confirm our studies in specimens of glioblastoma patients. Our data identify ZEB1 as a regulator of malignant glioma invasion, which is further corroborated by immunohistochemistry in specimens of invasive brain tumours of different grades. ZEB1 is associated with invasive glioma cells, with an increase in ZEB1-immunoreactive cell numbers in higher-grade tumours (Fig 6A). Since expression differences are difficult to interpret in immunoperoxidase staining, we performed immunofluorescence imaging for ZEB1, ROBO1 and N-cadherin in glioblastoma specimens. While not all glioblastomas stained for ZEB1, we found increased expression along the tumour invasion front (Fig 6B, Supporting Information Fig S6A), in accordance with our findings in xenografts (Fig 1C). Similarly, ROBO1 was found at the invasion front, and its expression was mutually exclusive to that of N-cadherin, which was preferentially at the tumour core (Fig 6C). To further corroborate the ZEB1 pathway in glioblastoma invasion and chemoresistance, we performed an immunoblot analysis in glioblastoma samples. ZEB1, N-cadherin, ROBO1, MGMT and Engrailed1 are frequently expressed in glioblastoma (Fig 6D, Supporting Information Fig S6B), confirming our experimental findings. Of note, ZEB1 was expressed in 40–45% of analysed samples. Immunofluorescent staining of patient tumour specimens revealed a 51 ± 9% overlap between ZEB1 and MGMT (Supporting Information Fig S6C), which corroborated the significant correlation between ZEB1 and MGMT indicated by Western analysis (Fig 6E, arrowheads). We also found, in accordance with reduced proliferation rates of ZEB1 expressing cells *in vitro* (Supporting Information Fig S2), that ZEB1 and the proliferation marker Ki-67 were mutually exclusive in patient specimens (3.66 ± 0.21%, Supporting Information Fig S6C).

Additionally, we found that protein expression of ZEB1, but surprisingly not MGMT, correlated with reduced survival of glioblastoma patients, as well as with shorter duration of successful TMZ therapy (Fig 6F). We found no significant correlation of ZEB1 with patient gender, age, or clinical performance (Supporting Information Fig S6D). However, when analysing changes in KPS scores over time, a slim but significant correlation with ZEB1 was observed (Supporting Information Fig S6D).

Glioblastoma has recently been stratified into different molecular subclasses (Phillips et al, 2006; Verhaak et al, 2010). Hence, we asked whether ZEB1 expression in these tumours is associated with any particular subclass. Immunoblot analysis of common pathway aberrations in these subclasses (Brennan et al, 2009) and other subclass markers (Phillips et al, 2006) revealed clustering of ZEB1 in the proliferative subclass, while ZEB1 negative tumours were slightly enriched for mesenchymal and proneural subclasses (Fig 6E). ZEB1 positive tumours showed a significant enrichment for PCNA and EGFR, while NF1 deletions were significantly more prevalent in ZEB1 negative specimens (Fig 6G, Supporting Information Fig S6E). Notably, induction of ZEB1 through EGF signalling (Ohashi et al, 2010) and correlation of EGFR expression and chemoresistance (Murat et al, 2008) has been described. Survival analysis was not significant for any subclass when stratifying tumour specimens into molecular subclasses, but in all cases ZEB1 negative tumours showed longer survival than ZEB1 positive specimens (Supporting Information Fig S6H). Additionally, the proliferative subclass demonstrated the shortest overall survival times, and the highest number of ZEB1 positive samples, further substantiating the link between ZEB1 and outcome.

Finally, the TCGA dataset (The Cancer Genome Atlas Research Network, 2008) supports the prognostic value of the ZEB1/miR-200/c-MYB pathway for glioblastoma, as well as significant co-occurrence of ZEB1, MGMT and ROBO1 in these tumours, and decreased levels of EGFR phosphorylation with reduced ZEB1 expression (Supporting Information Fig S6G and H).

## DISCUSSION

The findings presented here support that inducers of EMT in non-CNS cancers can promote single-cell invasion, chemoresistance and tumourigenesis in glioblastoma. While we identify a number of EMT-related factors in primary cell lines and tumour specimens, ZEB1 seems to dominate these processes, in up to 50% of patients with glioblastoma. However, the apparent heterogeneity of EMT-associated proteins in our samples indicates that these molecules may have redundant functions, fine-tune downstream pathways and/or serve specific purposes in a temporal and spatially regulated fashion.

Invasion is a common feature of brain malignancies from WHO grade II onwards, and we find ZEB1 expressed in invasive tumours (grade II–IV), but not in non-invasive neoplasms (grade I, data not shown). Thus, expression of ZEB1 seems to be associated with infiltrating tumour cells across a spectrum of glioma. A number of invasion pathways have been identified (Dirks, 2001; Hoelzinger et al, 2007; Lefranc et al, 2005), and it is likely that several contribute to glioma invasion. Invasive ZEB1-positive cells possess a greater degree of freedom for cellular locomotion due to the actions of ROBO1, uncoupling N-cadherin from the cytoskeleton (Rhee et al, 2002). This uncoupling allows N-cadherin to diffuse freely across the cell membrane, which contributes to the ability of invasive cells to break free from neighbouring tumour cells and infiltrate into the surrounding tissue. Tumour infiltration may further be facilitated by a variety of cellular migratory mechanisms (*e.g*. chemotaxis, autocrine/paracrine signalling, etc.).

In solid-tissue cancers outside the CNS, tumour invasion and EMT are driven by an E-cadherin to N-cadherin switch (Thiery et al, 2009), characterised by the loss of E-cadherin and a concomitant increase in N-cadherin. Here, we observe no significant levels of E-cadherin in our sample cohort, and also find no change in N-cadherin expression. In contrast, our data suggests that increased tumour invasion is mediated by a change in N-cadherin dynamics, mediated through ROBO1 by ZEB1. Of note, there are some cases of glioma where E-cadherin is present (Lewis-Tuffin et al, 2010). It is tempting to speculate that the subset of glioma in which E-cadherin is abundant has a more pronounced epithelial character, and might present as gliosarcoma. In these tumours, it is conceivable that the ‘classic’ E-to-N switch is the driving force of tissue infiltration.

Our data strongly support that invasive cells are also more resistant to the current standard of care drug TMZ, rendering these cells prime candidates for tumour recurrence. An intricate regulatory pathway, including miR-200c, c-MYB and MGMT, maintains this resistance. To date, *MGMT* promoter methylation is the most reliable prognostic marker for therapy resistance (Hegi et al, 2005). As ZEB1 does not influence methylation in our primary cell lines, it is possible that ZEB1 protein analysis may yield prognostic information that complement *MGMT* methylation data. ZEB1, miR-200c and c-MYB may constitute a novel pathway for the chemoresistance enzyme MGMT. We observed differences in the subcellular localisation of MGMT between xenografts (cytoplasmic) and clinical specimens (nuclear). Of note, Ishibashi et al. observed that MGMT is present in both fractions, and postulated that cytoplasmic MGMT is translocated to the nucleus after nuclear MGMT is depleted (Ishibashi et al, 1994). It is therefore possible that the nuclear staining pattern in patient specimens is due to previous treatment with alkylating agents, resulting in nuclear accumulation of MGMT. ZEB1 was a better predictor of outcome and therapy response at the protein level than MGMT. This may indicate that additional pathways regulate MGMT, as we found more specimens MGMT positive than ZEB1 positive. However, the strong correlation between ZEB1 and MGMT shows that ZEB1-positive cases are highly likely to express MGMT, explaining their poor response to TMZ.

In accordance with the cancer stem cell hypothesis, ZEB1 is linked to the expression of stemness-associated factors and tumourigenesis. Our data indicate that critical stem cell regulators, such as SOX2 and OLIG2, are induced by the ZEB1-miR-200 feedback loop in glioblastoma. The presence of ZEB1-positive cells at the tumour invasion front strongly supports an invasive niche that contains cancer stem cells (Cheng et al, 2011; Lathia et al, 2011). As the ZEB1-positive population is characterised by high motility and increased chemoresistance, these cancer stem cells are a candidate population for tumour recurrence. Of note, we have previously identified a more quiescent population of glioblastoma stem cells (Deleyrolle et al, 2011; Piccirillo et al, 2006), and we observed here that increased levels of ZEB1 are associated with slower proliferation. Whether the ZEB1 pathway directly reduces proliferation rates of glioblastoma stem cells remains to be tested.

Protein-level analysis shows that about 45% of human glioblastomas express ZEB1, and patients with ZEB1-negative glioblastomas have survival benefits that are likely related to an improved response to TMZ therapy. We cannot exclude that ZEB1 protein analysis is affected by sample collection site (*i.e*. core *vs*. edge), but fluorescence immunostaining detected ZEB1 only in samples that were found ZEB1 positive in immunoblots. Sample bias may also affect collections in databases (*e.g*. TCGA), which might explain the comparatively low prevalence of ZEB1 (and other EMT-related factors) in these datasets. Another possibility is post-transcriptional regulation of ZEB1 expression, *e.g*. through microRNAs, which may result in divergent levels of mRNA and protein. Further studies are required to address these issues.

ZEB1 expression in patient tumour samples is significantly enriched in the proliferative subclass (Brennan et al, 2009; Phillips et al, 2006), which appears in conflict with the lower proliferation of ZEB1-expressing cells in patient samples. A potential explanation is that ZEB1-positive cancer stem cells generate large numbers of rapidly dividing progenies, which in turn drive classification. Indeed, it has recently been observed that CD133-positive glioblastoma stem cells generate rapidly proliferating, but less invasive and less tumourigenic progenies (Chen et al, 2010). This study also found significantly lower percentages of CD133-positive cancer cells in mesenchymal *versus* proliferative glioblastoma.

An enrichment of EGFR amplification has been described in the proliferative subclass (Huse et al, 2011), which we confirmed in our cohort. Given the strong correlation with EGFR expression, it is conceivable that EGF signalling induces ZEB1 in these tumours. Since others have found induction of ZEB1 through beta-catenin or NF-KB signalling (Edwards et al, 2011; Kahlert et al, 2012), it is possible that different pathways activate ZEB1 in different subclasses. The enrichment of EGFR expression in ZEB1 positive tumours suggests that tumour treatment may be more efficacious via EGFR inhibition in combination with TMZ treatment. However, clinical trials employing this strategy have shown only limited effects to date (Wick et al, 2011). Further research is needed to determine whether better patient selection or new pharmacological approaches may be more successful and whether ZEB1 status may be of prognostic value in these cases.

Our findings establish ZEB1 as a regulator of invasion and chemoresistance in glioblastoma, and a candidate agent for tumour recurrence. The multiple ZEB1-associated regulators of brain tumour growth and invasion outlined in this study provide potential targets for future therapeutic approaches intervening at the level of invasion and/or chemoresistance.

## MATERIALS AND METHODS

### Cell culture

Tumour cell lines were generated (Piccirillo et al, 2006) and maintained (Siebzehnrubl et al, 2009) as described. Briefly, 50,000 cells were seeded per ml of culture medium (N2, Invitrogen, Carlsbad, CA) in the presence of mitogens (20 ng/ml each of EGF and FGF2, Sigma, St. Louis, MO). Cells were propagated as spheres and passaged using Accutase (PAA, Cölbe, Germany) every 7 days. For experiments with adherent cells, spheres were dissociated and plated in N2 medium supplemented with 1% foetal bovine serum (FBS). For scratch assays, 2 × 10^6^ cells were plated per well of a six-well culture plate coated with poly-L-ornithine and laminin (15 µg/ml) in N2 containing 1% FBS, and grown to confluence overnight. Confluent monolayers were scratched with a pipette tip, and imaged at the time of the lesion and 24 h later. For sphere formation assays, 1,000 cells were plated per well into 96-well culture plates in N2 containing EGF and bFGF. Spheres larger than 50 µm were counted 7 days after plating.

### Flow cytometry

Immunostaining of cancer cells and flow cytometry was performed as described (Deleyrolle et al, 2011; Piccirillo et al, 2006). Briefly, live cells were dissociated using PBS and 0.5 mM EDTA and subsequently incubated with primary antibody or IgG controls in PBS containing 0.1% BSA for 1 h on ice. Following 2 washes in PBS, cells were analysed on a BD LSR II (BD Biosciences, San Jose, CA). Data was analysed and dot plots were generated using FlowJo Ver. 8.8.7 (Tree Star, Ashland, OR).

### Cell viability assay

The Methyltetrazolium bromide (MTT) assay was used as indicator of cell viability and performed as described (Holsken et al, 2006). Briefly, 10,000 cells were plated per well into 96-well cell culture plates and treated 1 h after plating with varying concentrations of TMZ (ranging 5 µM–5 mM, Tocris, Ellisville, MO). Concentration-effect curves for TMZ treatment were generated by nonlinear regression analysis as described (Holsken et al, 2006). Bar graphs are derived from individual concentration measurements, compared to the appropriate controls.

### Knock-down experiments

Plasmids for knockdown of ZEB1 and expression of hsa-miR-200c, as well as antago-miR-200c and control sequences are as described previously (Wellner et al, 2009). Plasmids for knockdown of c-MYB, MGMT and ROBO1 were obtained from OpenBiosystems (Lafayette, CO). The expression plasmid for c-MYB (Clarke et al, 1988) was a kind gift of Dr. J.S. Lipsick (Stanford University). Expression plasmids for MGMT and ZEB1 were obtained from Origene (Rockville, MD). Cancer cells were transfected using Lipofectamine LTX (Invitrogen) according to the manufacturer's instructions. Transfected cells were selected using puromycin or geneticin (Sigma) before being used for subsequent experiments.

### Animal experiments

Adult female Fox-Chase SCID mice (Charles River, Wilmington, MA) were used for *in vivo* tumour transplants. All procedures were performed according to NIH and institutional guidelines for animal care and handling. After animals were deeply anaesthetised using USP grade Isoflurane (Halocarbon, North Augusta, SC), an incision was made in the scalp, the skull demonstrated and a hole drilled at the coordinates Bregma −0.5 mm anterior and −1.5 mm lateral. A Hamilton syringe was lowered 2.5 mm into the burr hole, and 1 µl of a cell suspension was injected over 5 min before the needle was retracted. After the incision was closed with surgical staples the animal was allowed to recover before being returned to the cage. Animals were transplanted with doses ranging from 1,000 to 100,000 cells, and tumour-bearing animals were scored regularly for tumour-related symptoms. Moribund animals were anaesthetised and transcardially perfused with 4% paraformaldehyde in saline, the brains removed, postfixed and prepared for histology. For *in vivo* TMZ treatment, animals received orthotopic grafts of 150,000 cells. One week after transplantation, tumour-bearing animals were intraperitoneally injected with 20 mg/kg TMZ in saline (final DMSO concentration 25%). Animals received five injections over 5 days, corresponding to one cycle of TMZ treatment. Animals at endpoint (defined as bodyweight loss ≥20% or observation of severe neurological symptoms) were perfused and tumour presence was confirmed histologically. For *in vivo* analysis of inducible ROBO1 knockdown, animals received orthotopic grafts of 150,000 cells. One group received doxycycline injections i.p. (10 mg/kg in saline) at day 4 post surgery and then every other day.

### Patient sample collection

Tissue specimens from glioblastoma patients were obtained from the Florida Center for Brain Tumour Research (FCBTR) and the UF and Shands Department of Pathology with approval from the UF Institutional Review Board (IRB). Informed consent was obtained from all subjects and all experiments conformed to the principles set out in the WMA Declaration of Helsinki and the NIH Belmont Report.

### Immunohistochemistry and Immunocytochemistry

Immunostainings were performed as described (Siebzehnrubl et al, 2009; Zheng et al, 2006). Paraffin-embedded patient material was deparaffinised, followed by heat-mediated antigen retrieval in a 10 mM citric acid buffer (pH 6.0) or Trilogy (Cell Marque, Rocklin, CA), and tissue was subsequently immunostained with 3,3′-diaminobenzidine (Vector Elite Kit, Vector labs, Burlingame, CA) or fluorescence-conjugated antibodies using standard protocols. A table of employed antibodies, suppliers and dilutions can be found in the Supporting Information.

### Image acquisition and data analysis

Low-power fluorescent images were taken on a Leica DMLB epifluorescence microscope (Bannockburn, IL) equipped with a CCD camera (Spot Imaging Solutions, Sterling Heights, MI). To obtain full images of brain sections, multiple grey-scale images were acquired per section using Spot Advanced software (Spot Imaging Solutions) and merged into a full image and inverted into black-on-white images using Photoshop CS4 (Adobe Systems, San Jose, CA). Photomerged images were imported into ImageJ and threshold levels were adjusted to distinguish tumour from background. Using the wand tool, all outlines of positively stained (black) tumour areas were selected in each section and the perimeter (line surrounding the tumour) and area of the tumour were measured. The wand tool allows an exact distinction between black (tumour) and white (parenchyma) regions; hence, the measurement of tumour outline and area is unbiased. The ratio of the squared perimeter distance over the area (*P*^2^/*A*) was calculated and used to compare invasive properties of different tumours. Since *P*^2^/*A* is a dimensionless number, the resulting figure is termed ‘invasion index’. A higher invasion index is indicative of a more dissociated tumour, whereas a lower invasion index represents a more spherical tumour. High-power images were taken on an Olympus BX-81 DSU spinning disc confocal microscope (Olympus, Center Valley, PA) and projection images of *z*-stacks were generated using Slidebook (Olympus) software. For mean fluorescence intensity analysis, two visual fields within the tumour core or edge were selected at random per animal, and a confocal *z*-stack through the entire section was obtained. For each stack, one plane of section was selected and mean gray values for each channel obtained using ImageJ, and the ratio of average channel intensities for ZEB1 and Hoechst was calculated.

### RNA isolation and quantitative real-time PCR

Total RNA was isolated from tumour sphere or adherent cultures using the RNeasy Mini Kit (Qiagen, Valencia, CA) according to the manufacturer's instructions. RNA was quantified on a Nanodrop Spectrophotometer (Thermo, Wilmington, DE), and 1 µg of total RNA was used for cDNA synthesis as described (Siebzehnrubl et al, 2009). Twenty-five nanograms of cDNA were used for quantitative PCR using the SYBR green PCR master mix (Applied Biosystems, Carlsbad, CA) on an ABI 7900HT (Applied Biosystems) as previously described (Siebzehnrubl et al, 2009). Expression levels of ZEB1 were quantified in triplicate relative to beta-actin using the ΔΔ*C*_t_ method. Primer sequences and amplification times are described elsewhere (Wellner et al, 2009).

### Protein isolation and Western blotting

Proteins were isolated from cancer cell cultures and primary tumour specimens as described (Siebzehnrubl et al, 2009). For Western blotting, 5–40 µg of denatured protein were loaded on 4–12% Bis–Tris reducing gel (Invitrogen), separated and blotted onto a PVDF membrane (iBlot, Invitrogen). Blots were blocked and probed with respective primary and secondary antibodies (see Supporting Information) as described (Siebzehnrubl et al, 2009), and developed using the ECL Plus kit (Amersham, Piscataway, NJ) on a FluorChemQ Multi Image III (Cell Biosciences, Santa Clara, CA) and AlphaInnotech software version 1.0.1.1. Band densitometry was performed using ImageJ.

### Chromatin immunoprecipitation

Chromatin was cross-linked and isolated from 2.0 × 10^7^ cells in sphere culture using the Simple ChIP kit (Cell Signaling, Danvers, MA) according to the manufacturer's instructions. Following immunoprecipitation with anti-MYB and IgG control antibodies, the MGMT promoter region was amplified with specific primers (primer sequences and PCR conditions available upon request).

### Bisulfite genomic sequencing

DNA was prepared and bisulfite converted as previously described (Pardo et al, 2011). Bisulfite converted DNA was amplified using *MGMT*-specific primers (sequences available upon request). PCR products were gel-extracted and cloned into pGEM T-easy vector (Promega, Madison, WI) followed by transformation of TOP10 cells (Invitrogen). Individual clones were sequenced and data was analysed using Sequencher for sequence alignment and MethylViewer to assign site-specific methylation status (Pardo et al, 2011). Molecules with <97% conversion efficiency were excluded.

### Statistical testing

Statistical analyses were performed in GraphPad Prizm 5.0 (GraphPad Software, La Jolla, CA). Statistical tests are indicated in the text. In all analyses, a *p*-value < 0.05 was deemed significant. We used the D'Agostino–Pearson test for normal distribution of values. A Bonferroni Multiple Comparison test was applied to all ANOVA analyses post-test. Observers were blinded to the patient data (including survival time) when performing tumour sample protein analyses. We used the R statistical software package (V. 2.15.0) to calculate descriptive statistics, create graphics and perform all analyses of patient specimens. To evaluate the possible associations between molecular subclass (mesenchymal, proneural or proliferative) and the other variables in the study (age, gender, mortality, KPS, ZEB1 and MGMT), we used chi-square, Fisher's exact or Kruskal–Wallis tests, as appropriate. We used the Kaplan–Meier method to perform survival analyses comparing groups classified by ZEB1 or MGMT level, and to create survival plots. To estimate the effects of ZEB1 and MGMT when controlling for age and gender, we used Cox Proportional Hazards models. In all survival analyses, the outcome variable was time from start of treatment until death. Subjects still alive at the time or analysis and subjects lost to follow-up were considered censored.

Glioblastoma has a very poor prognosis, mainly due to its infiltrating and therapy resistant cells, which eventually lead to tumor recurrence. Outside the CNS, epithelial-mesenchymal transition has been identified as an inducer of invasion, chemoresistance and metastasis in solid tissue cancers, but the extent to which similar processes affect brain cancer progression is currently unknown.

RESULTS:

We find that in glioblastoma the processes of tumor formation, invasion and chemoresistance are not separate entities, but intertwined. The EMT activator ZEB1 is a master regulator of all three elements, indicating that EMT-associated factors are involved in brain tumor progression. Additional regulatory molecules mediate the individual parts of this triad. Tumor formation and stemness are associated with increased expression of SOX2 and OLIG2; ROBO1 elicits redistribution of N-cadherin, which reduces cell-cell adhesion and affords greater migratory capacity; c-MYB increases expression of the chemoresistance enzyme MGMT. Thus, ZEB1-positive tumor cells evade conventional anti-cancer therapy and are a potential source for tumor recurrence. Importantly, glioblastoma patients with detectable levels of ZEB1 have a poorer prognosis and respond less to Temozolomide treatment than patients where ZEB1 is undetectable.

IMPACT:

Our findings indicate that (i) a common pathway drives tumorigenesis, tissue invasion, and chemoresistance in glioblastoma; and (ii) ZEB1 may have prognostic value in glioblastoma patients.

## Author contributions

FAS planned and performed experiments, analysed data and wrote the manuscript. DJS, LPD and BT performed experiments and contributed to the manuscript. DS, OS, and MDK performed experiments. KGD and ATY provided neuropathological specimens and diagnoses. DN performed statistical analyses and interpreted statistical data. SB and TB provided hsa-miR-200c, shGFP and shZEB1 vectors and contributed to the manuscript. NHN and MPK performed and interpreted MGMT methylation analysis. BAR, MRS and DAS were involved in discussions during the course of this study and contributed to the manuscript.

## Supporting Information

As a service to our authors and readers, this journal provides supporting information supplied by the authors. Such materials are peer reviewed and may be re-organized for online delivery, but are not copy-edited or typeset. Technical support issues arising from supporting information (other than missing files) should be addressed to the authors.

**Figure S1. (A) SNAI1, SNAI2 and E**-**cadherin are expressed in hGBM L0, but not in the other two lines.** (**B**) ZEB1 is the only EMT-associated factor analyzed that affects outcome in the TCGA dataset (REF). (**C**) ZEB1 is increased at the tumor edge of hGBM L1. Scale bars 10 µm. (**D**) Tumor mass cells (asterisks) are characterized by membrane-associated beta-catenin, which is absent in invasive cells. Note that invasive cells show no nuclear accumulation of beta-catenin (arrowheads). Scale bars 10 µm. **Figure S2. (A) Beta-catenin is associated with the cell membrane in both control cells (shGFP) and ZEB1 knockdown, while only controls are positive for ZEB1.** In addition, control cells appear more dispersed after plating. Scale bars 10 µm. (**B**) ZEB1 knockdown reduces ZEB1 mRNA levels in tumor-sphere cultures (representative data from hGBM L1, *n* = 3, *t*-test). ZEB1 knockdown increases cellular proliferation approximately twofold, whereas forced expression decreases proliferation rates (hGBM L0, *n* = 3, one-way ANOVA). (**C**) Threshold images generated from immunofluorescence staining depicted in Figure 2B. These images are used for measuring invasion indices. (**D**) Putative miR-200c binding sites in the 5′-UTR of c-MYB, SOX2, OLIG2 and CD133. **Figure S3. (A) ROBO1 is preferentially localized at the cell membrane of control cells (shGFP), and absent in ZEB1 knockdown cells. Scale bar, 10** **µm.** (**B**) ROBO1 expression is increased by antagonizing miR-200 in ZEB1 knockdown cells, and decreased by forced expression of miR-200 in ZEB1 overexpressing cells. (**C**) Antagonizing miR-200 increases cell migration (*n* = 3; Mann–Whitney *U*-test). (**D**) ROBO1 knockdown reduces migration in a scratch assay (*n* = 3, Mann–Whitney *U*-test). (**E**) ROBO1 knockdown can be rescued by a non-targeted ROBO1 construct. (**F**) ROBO1 knockdown reduces tumor invasion *in vivo* (*n* = 5 animals each, one-way ANOVA). (**G**) Forced expression of ROBO1 rescues cell migration in ZEB1 knockdown cells, while ROBO1 knockdown reduces migration in ZEB1 overexpressing cells (*n* = 3; Mann–Whitney *U*-test). **Figure S4. (A) Antagonizing miR-200 increases expression of c-MYB and MGMT, as well as chemoresistance in ZEB1 knockdown cells (*n*** **=** **8, one-way ANOVA).** (**B**) Forced expression of miR-200 reduces expression of c-MYB and MGMT in ZEB1 overexpressing cells, and results in reduced chemoresistance (*n* = 8, one-way ANOVA). (**C**) Knockdown of c-MYB reduces expression of MGMT, while overexpression of c-MYB increases MGMT levels. Both have no influence on ZEB1 expression. Resistance to TMZ is significantly reduced by c-MYB knockdown *in vitro* (*n* = 8, oneway ANOVA). (**D**) Chemoresistance in ZEB1-knockdown cells can be rescued by expression of c-MYB (*n* = 8, one-way ANOVA). (**E**) Knockdown of c-MYB reduces expression of MGMT and chemoresistance in ZEB1-overexpressing cells. (F) Rescue of MYB, MGMT and ZEB1 knockdown with respective untargeted constructs. **Figure S5. (A) Bisulfite genomic sequencing of the *MGMT* promoter demonstrates no significant methylation changes in ZEB1 knockdown cells.** The *MGMT* promoter was amplified from −555 to +120 relative to the TSS. The frequency of methylation at each CG site was calculated from at least 10 cloned and sequenced molecules for each condition. Data is presented as the average methylation frequency and the position of each CG site relative to the TSS is indicated. (**B**) ZEB1 knockdown increases survival of tumor-bearing animals after one cycle (five injections) of TMZ treatment *in vivo* (log-rank test). Expression of c-MYB in ZEB1 knockdown cells abolished this survival benefit (log-rank test). **Figure S6. (A) ZEB1 negative glioblastoma specimens do not stain for ZEB1 at the tumor invasion front. Scale bar 20** **µm (applies to all images).** (**B**) ZEB1, E-cadherin, SNAI1, SNAI2, and Twist-1 are not expressed in glioblastoma specimens. (**C**) ZEB1 and the proliferation marker Ki-67 are mutually exclusive in tumor specimens (3.66 ± 0.21% co-localization; *n* = 14; Venn diagrams illustrate overlapping populations of ZEB1 and Ki-67, and ZEB1 and MGMT, respectively). (**D**) Neither age, nor KPS scores (post-OP or last recorded) correlate with ZEB1, but a significant correlation was found for the ratio of last recorded to post-OP KPS score. (**E**) PCNA and EGFR are significantly enriched in ZEB1 positive tumor specimens, while loss of NF1 is significantly more frequent in ZEB1 negative samples (Fisher's exact test). (**F**) Survival analysis for ZEB1 class by molecular subgroups revealed no significant outcome benefit in either subgroup, but shows a general trend for improved outcome in ZEB1 negative tumors. Note the shorter overall survival in the proliferative subgroup (which has the highest percentage of ZEB1+ specimens). (**G**) TCGA analysis of the ZEB1/miR-200c/c-MYB pathway shows significant changes in patient outcome. Both MGMT and ROBO1 frequently co-occur with ZEB1 in TCGA samples (*p*-values are from Fisher's exact test). (**H**) ZEB1 positive samples show higher levels of p-EGFR in RPPA. **Table S1.** Clinical information of patient samples used in this study. **Table S2.** Vendor information for antibodies used in this study.
