# Supplementary material for: The ZEB1 pathway links glioblastoma initiation, invasion and chemoresistance
Source: EMBO Mol Med. 2013 Jul 1;5(8):1196–212. doi: 10.1002/emmm.201302827 (PMC3944461; doi:10.1002/emmm.201302827)
Supplement: Supplementary file 1 — Figure S1. (A) SNAI1, SNAI2 and E-cadherin are expressed in hGBM L0, but not in the other two lines. (B) ZEB1 is the only EMT-associated factor analyzed that affects outcome in the TCGA dataset (REF). (C) ZEB1 is increased at the tumor edge of hGBM L1. Scale bars 10 µm. (D) Tumor mass cells (asterisks) are characterized by membrane-associated beta-catenin, which is absent in invasive cells. Note that invasive cells show no nuclear accumulation of beta-catenin (arrowheads). Scale bars 10 µm. Figure S2. (A) Beta-catenin is associated with the cell membrane in both control cells (shGFP) and ZEB1 knockdown, while only controls are positive for ZEB1. In addition, control cells appear more dispersed after plating. Scale bars 10 µm. (B) ZEB1 knockdown reduces ZEB1 mRNA levels in tumor-sphere cultures (representative data from hGBM L1, n = 3, t-test). ZEB1 knockdown increases cellular proliferation approximately twofold, whereas forced expression decreases proliferation rates (hGBM L0, n = 3, one-way ANOVA). (C) Threshold images generated from immunofluorescence staining depicted in Figure 2B. These images are used for measuring invasion indices. (D) Putative miR-200c binding sites in the 5′-UTR of c-MYB, SOX2, OLIG2 and CD133. Figure S3. (A) ROBO1 is preferentially localized at the cell membrane of control cells (shGFP), and absent in ZEB1 knockdown cells. Scale bar, 10 µm. (B) ROBO1 expression is increased by antagonizing miR-200 in ZEB1 knockdown cells, and decreased by forced expression of miR-200 in ZEB1 overexpressing cells. (C) Antagonizing miR-200 increases cell migration (n = 3; Mann–Whitney U-test). (D) ROBO1 knockdown reduces migration in a scratch assay (n = 3, Mann–Whitney U-test). (E) ROBO1 knockdown can be rescued by a non-targeted ROBO1 construct. (F) ROBO1 knockdown reduces tumor invasion in vivo (n = 5 animals each, one-way ANOVA). (G) Forced expression of ROBO1 rescues cell migration in ZEB1 knockdown cells, while ROBO1 knockdown reduces migration in ZEB1 [file emmm0005-1196-SD1.pdf]

Supplemental Information

The ZEB1 pathway links glioblastoma initiation, invasion and chemoresistance

FA Siebzehnrubl, DJ Silver, B Tugertimur, LP Deleyrolle, D Siebzehnrubl, MR Sarkisian, KG Devers, AT Yachnis, MD Kupper, D Neal, NH Nabilsi, MP Kladde, O Suslov, S Brabletz, T Brabletz, BA Reynolds, DA Steindler

Table of contents:

Figure S1	EMT factors in glioblastoma	p. 2
Figure S2	Additional ZEB1 data; microRNA binding sites	p. 3
Figure S3	ROBO1 mediates invasion	p. 4
Figure S4	c-MYB and MGMT mediate chemoresistance	p. 5
Figure S5	MGMT promoter methylation; additional K-M curves	p. 6
Figure S6	Additional patient and TCGA data	p. 7
Table S1	Clinical information of patient samples	p. 9
Table S2	Vendor information for antibodies	p. 10

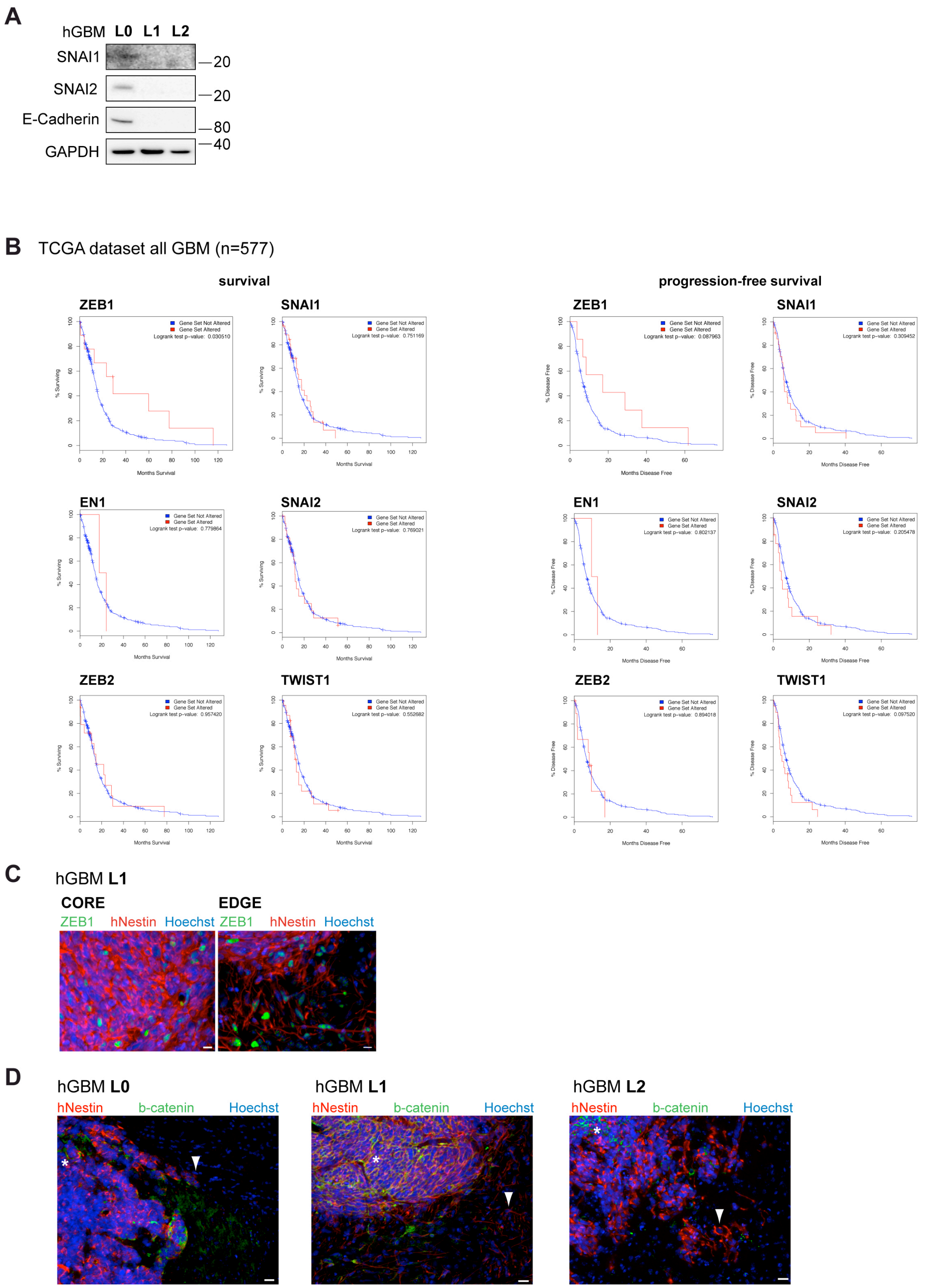

Figure S1

(A) SNAI1, SNAI2, and E-cadherin are expressed in hGBM L0, but not in the other two lines. (B) ZEB1 is the only EMT-associated factor analyzed that affects outcome in the TCGA dataset (REF). (C) ZEB1 is increased at the tumor edge of hGBM L1. Scale bars 10 µm. (D) Tumor mass cells (asterisks) are characterized by membrane-associated beta-catenin, which is absent in invasive cells. Note that invasive cells show no nuclear accumulation of beta-catenin (arrowheads). Scale bars 10 µm.

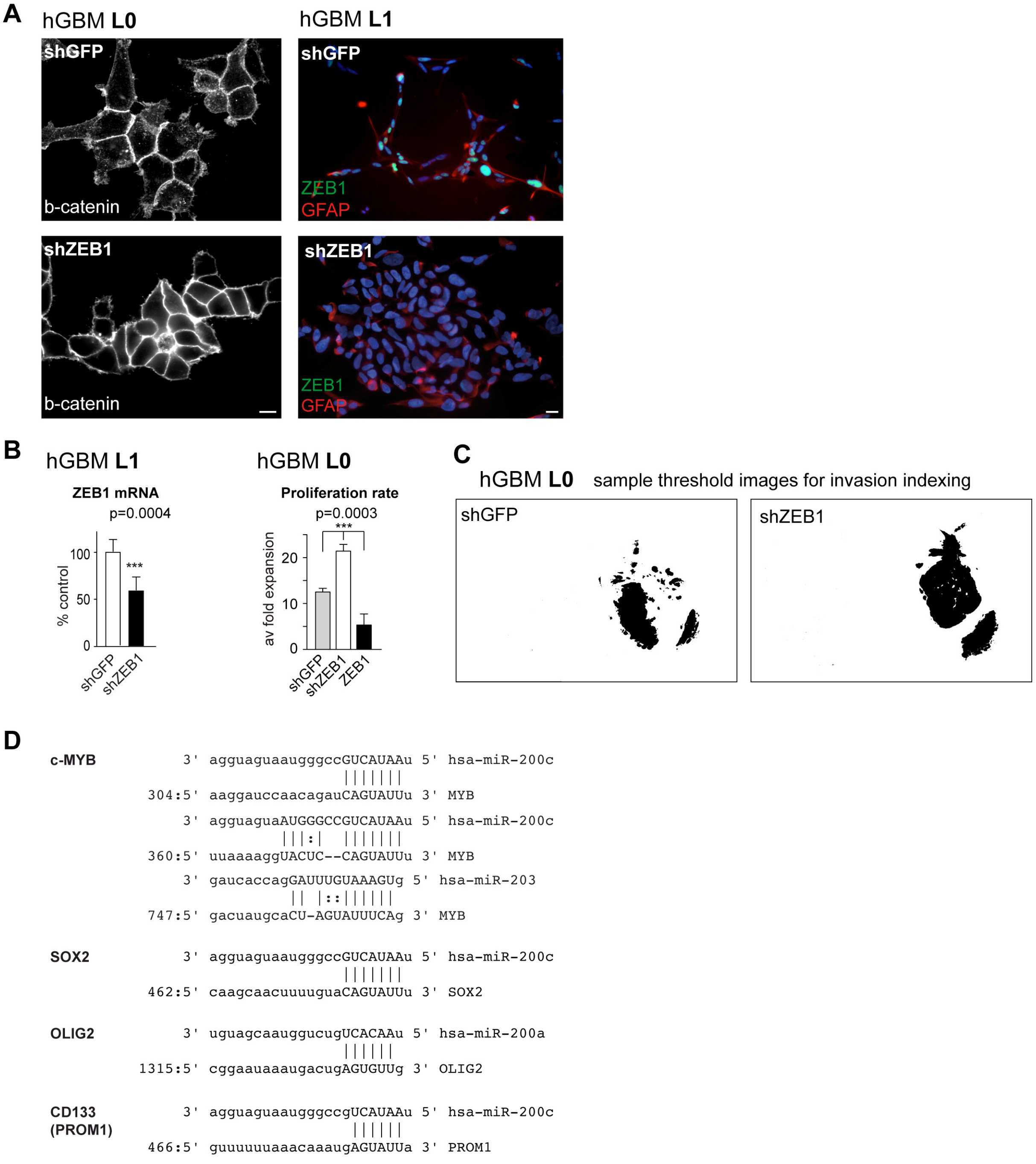

Figure S2

(A) Beta-catenin is associated with the cell membrane in both control cells (shGFP) and ZEB1 knockdown, while only controls are positive for ZEB1. In addition, control cells appear more dispersed after plating. Scale bars 10 µm. (B) ZEB1 knockdown reduces ZEB1 mRNA levels in tumor-sphere cultures (representative data from hGBM L1, n=3, t-test). ZEB1 knockdown increases cellular proliferation approximately two-fold, whereas forced expression decreases proliferation rates (hGBM L0, n=3, one-way ANOVA). (C) Threshold images generated from immunofluorescence staining depicted in Figure 2B. These images are used for measuring invasion indices. (D) Putative miR-200c binding sites in the 5'-UTR of c-MYB, SOX2, OLIG2 and CD133.

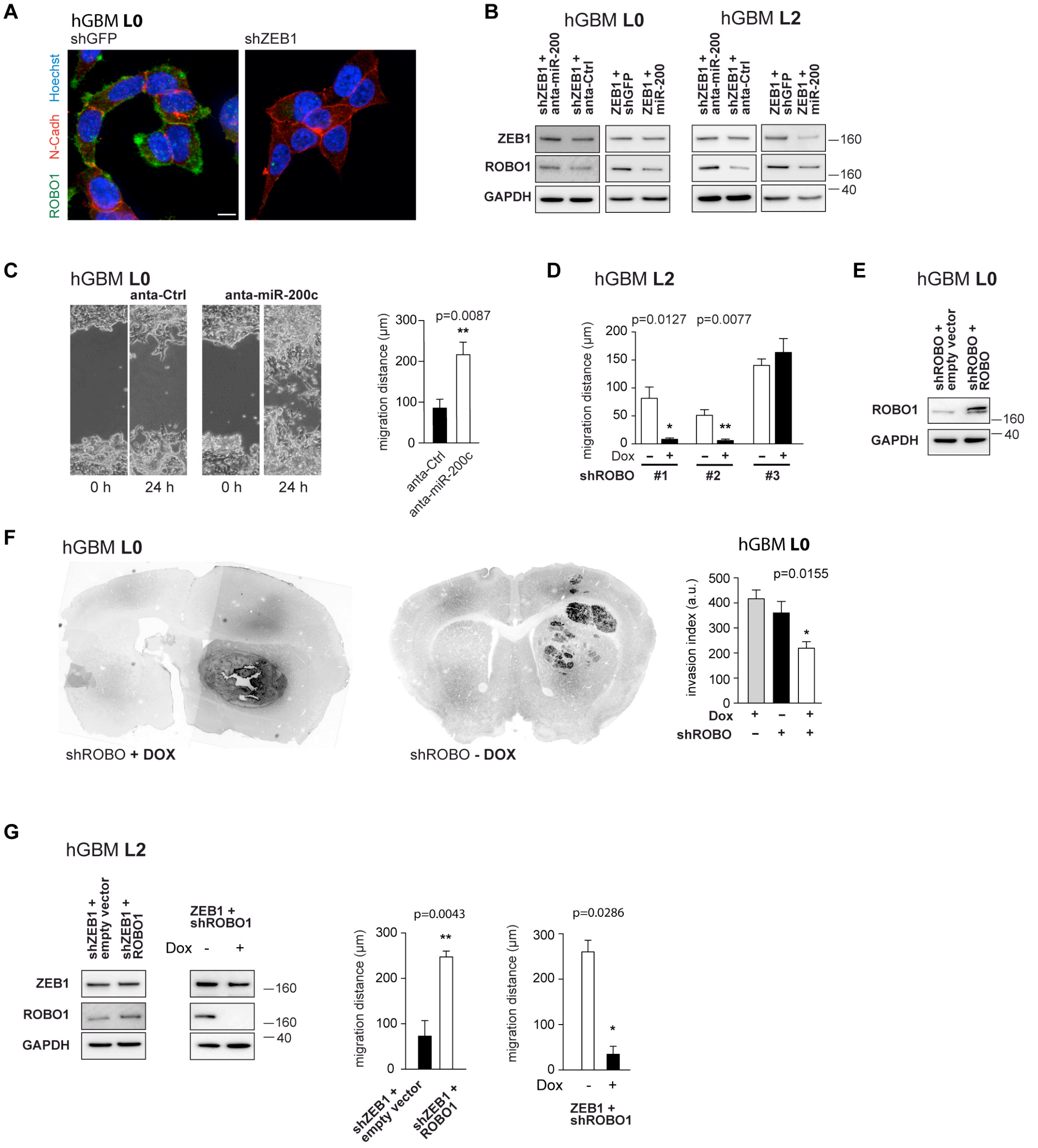

Figure S3

(A) ROBO1 is preferentially localized at the cell membrane of control cells (shGFP), and absent in ZEB1 knockdown cells. Scale bar, 10 µm. (B) ROBO1 expression is increased by antagonizing miR-200 in ZEB1 knockdown cells, and decreased by forced expression of miR-200 in ZEB1 overexpressing cells. (C) Antagonizing miR-200 increases cell migration (n=3; Mann-Whitney U test). (D) ROBO1 knockdown reduces migration in a scratch assay (n=3, Mann-Whitney U test). (E) ROBO1 knockdown can be rescued by a non-targeted ROBO1 construct. (F) ROBO1 knockdown reduces tumor invasion *in vivo* (n=5 animals each, one-way ANOVA). (G) Forced expression of ROBO1 rescues cell migration in ZEB1 knockdown cells, while ROBO1 knockdown reduces migration in ZEB1 overexpressing cells (n=3; Mann-Whitney U test).

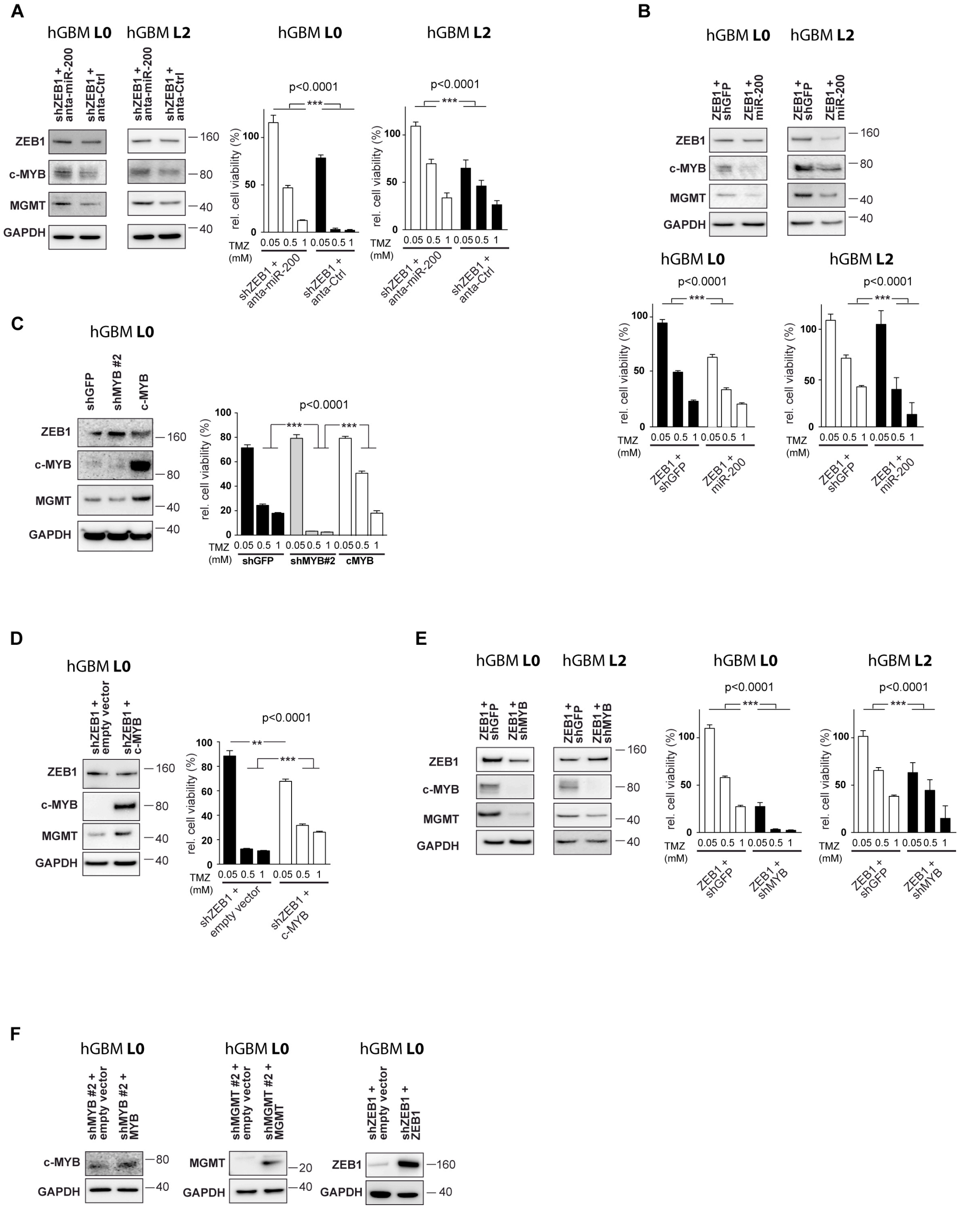

Figure S4

(A) Antagonizing miR-200 increases expression of c-MYB and MGMT, as well as chemoresistance in ZEB1 knockdown cells (n=8, one-way ANOVA). (B) Forced expression of miR-200 reduces expression of c-MYB and MGMT in ZEB1 overexpressing cells, and results in reduced chemoresistance (n=8, one-way ANOVA). (C) Knockdown of c-MYB reduces expression of MGMT, while overexpression of c-MYB increases MGMT levels. Both have no influence on ZEB1 expression. Resistance to TMZ is significantly reduced by c-MYB knockdown *in vitro* (n=8, one-way ANOVA). (D) Chemoresistance in ZEB1-knockdown cells can be rescued by expression of c-MYB (n=8, one-way ANOVA). (E) Knockdown of c-MYB reduces expression of MGMT and chemoresistance in ZEB1-overexpressing cells. (F) Rescue of MYB, MGMT and ZEB1 knockdown with respective untargeted constructs.

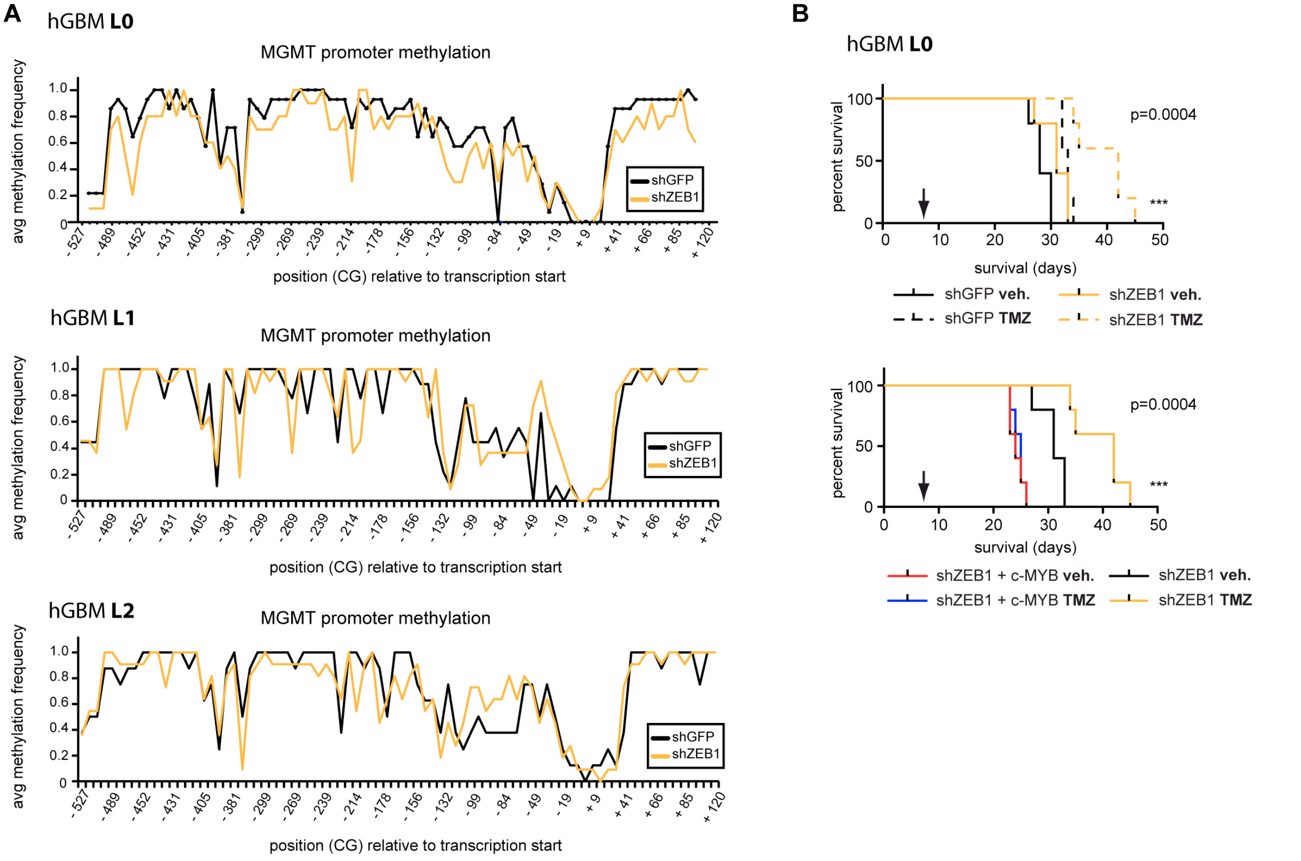

Figure S5

(A) Bisulfite genomic sequencing of the *MGMT* promoter demonstrates no significant methylation changes in ZEB1 knockdown cells. The *MGMT* promoter was amplified from -555 to +120 relative to the TSS. The frequency of methylation at each CG site was calculated from at least 10 cloned and sequenced molecules for each condition. Data is presented as the average methylation frequency and the position of each CG site relative to the TSS is indicated. (B) ZEB1 knockdown increases survival of tumor-bearing animals after one cycle (5 injections) of TMZ treatment *in vivo* (log-rank test). Expression of c-MYB in ZEB1 knockdown cells abolished this survival benefit (log-rank test).

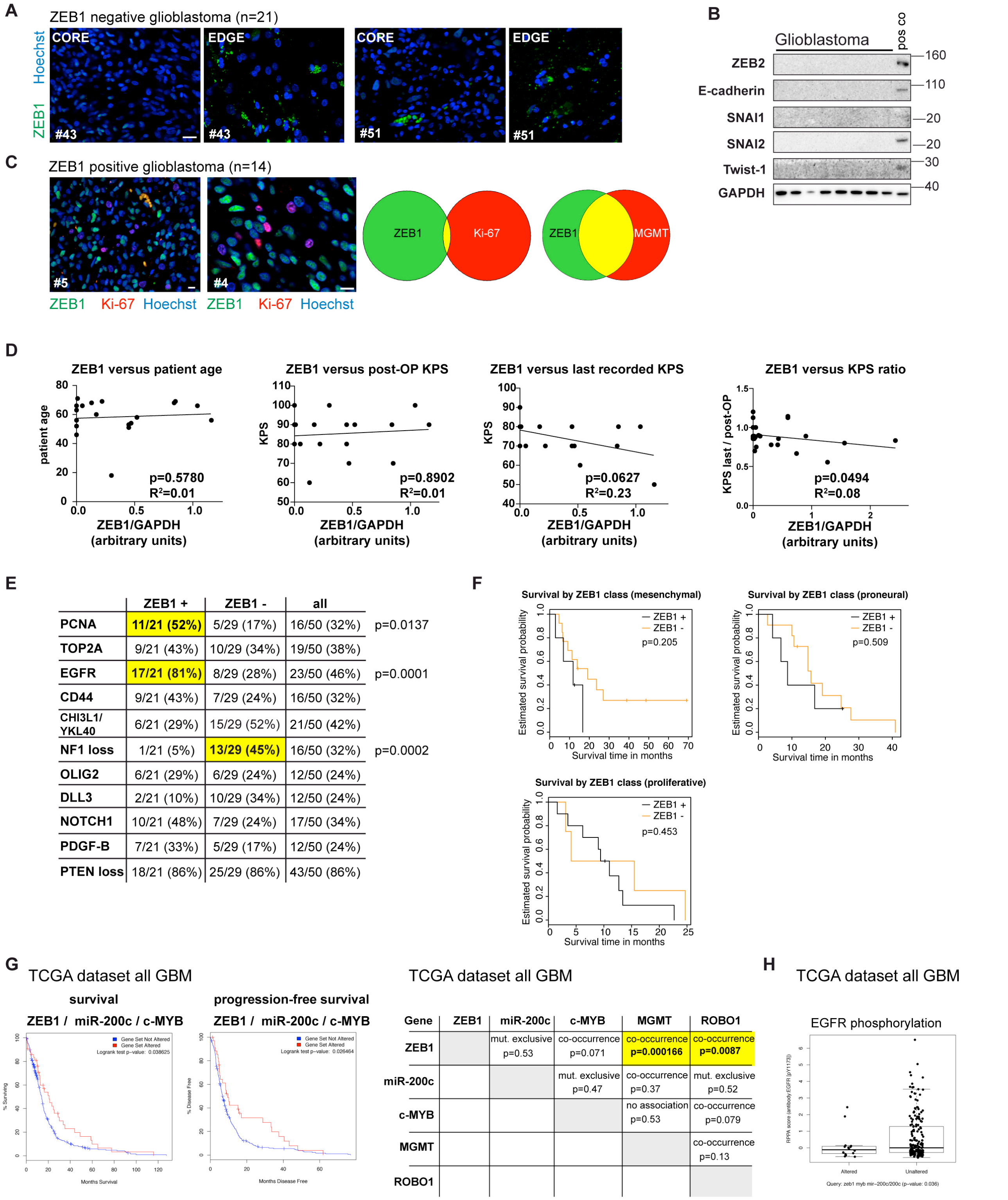

	ZEB1 +	ZEB1 -	all	
PCNA	**11/21 (52%)**	5/29 (17%)	16/50 (32%)	p=0.0137
TOP2A	9/21 (43%)	10/29 (34%)	19/50 (38%)	
EGFR	**17/21 (81%)**	8/29 (28%)	23/50 (46%)	p=0.0001
CD44	9/21 (43%)	7/29 (24%)	16/50 (32%)	
CHI3L1/ YKL40	6/21 (29%)	15/29 (52%)	21/50 (42%)	
NF1 loss	1/21 (5%)	**13/29 (45%)**	16/50 (32%)	p=0.0002
OLIG2	6/21 (29%)	6/29 (24%)	12/50 (24%)	
DLL3	2/21 (10%)	10/29 (34%)	12/50 (24%)	
NOTCH1	10/21 (48%)	7/29 (24%)	17/50 (34%)	
PDGF-B	7/21 (33%)	5/29 (17%)	12/50 (24%)	
PTEN loss	18/21 (86%)	25/29 (86%)	43/50 (86%)	

Gene	ZEB1	miR-200c	c-MYB	MGMT	ROBO1
ZEB1		mut. exclusive p=0.53	co-occurrence p=0.071	co-occurrence **p=0.000166**	co-occurrence **p=0.0087**
miR-200c			mut. exclusive p=0.47	co-occurrence p=0.37	mut. exclusive p=0.52
c-MYB				no association p=0.53	co-occurrence p=0.079
MGMT					co-occurrence p=0.13
ROBO1					

Figure S6

(A) ZEB1 negative glioblastoma specimens do not stain for ZEB1 at the tumor invasion front. Scale bar 20 µm (applies to all images). (B) ZEB1, E-cadherin, SNAI1, SNAI2, and Twist-1 are not expressed in glioblastoma specimens. (C) ZEB1 and the proliferation marker Ki-67 are mutually exclusive in tumor specimens (3.66 ± 0.21 % co-localization; n=14; Venn diagrams illustrate

overlapping populations of ZEB1 and Ki-67, and ZEB1 and MGMT, respectively). (D) Neither age, nor KPS scores (post-OP or last recorded) correlate with ZEB1, but a significant correlation was found for the ratio of last recorded to post-OP KPS score. (E) PCNA and EGFR are significantly enriched in ZEB1 positive tumor specimens, while loss of NF1 is significantly more frequent in ZEB1 negative samples (Fisher's exact test). (F) Survival analysis for ZEB1 class by molecular subgroups revealed no significant outcome benefit in either subgroup, but shows a general trend for improved outcome in ZEB1 negative tumors. Note the shorter overall survival in the proliferative subgroup (which has the highest percentage of ZEB1+ specimens). (G) TCGA analysis of the ZEB1/miR-200c/c-MYB pathway shows significant changes in patient outcome. Both MGMT and ROBO1 frequently co-occur with ZEB1 in TCGA samples (p-values are from Fisher's exact test). (H) ZEB1 positive samples show higher levels of p-EGFR in RPPA.

Table S1: Clinical information of patient samples used in this study.

Patient #	age (yr)	sex	diagnosis	survival (m)	TMZ	TMZ duration (m)	TMZ response	ZEB1	IDH1	PDGF-B	Notch1	Olig2	DLL3	PTEN	PCNA	TOP2A	EGFR	NF1	CD44	CHI3L1	subclass
1	69	M	glioblastoma	8.7	un	na	na	+	-	-	+	+	-	-	-	-	+	+	-	-	PN
4	56	M	glioblastoma	2.9	un	na	na	+	-	-	+	+	-	-	+	-	+	+	+	+	MES
5	68	M	glioblastoma	13.4	y	4	progression	+	-	-	-	-	-	-	+	-	+	+	+	-	PROL
6	78	M	glioblastoma	6.2	un	na	na	+	-	-	-	-	-	-	-	+	+	+	+	-	PROL
7	66	F	glioblastoma	16.8	n	na	progression	+	-	+	+	+	-	-	+	-	+	+	-	-	PN
8	78	M	glioblastoma	11	un	na	na	+	-	-	+	-	-	-	+	+	+	+	+	+	PROL
14	43	M	glioblastoma	9	y	2	progression	+	-	-	+	-	-	-	+	-	+	+	-	-	PROL
15	74	F	glioblastoma	22.7	y	un	progression	+	-	-	+	-	-	-	+	-	+	+	-	-	PROL
20	56	F	glioblastoma	11.8	y	3.25	progression	+	-	-	+	-	-	-	-	+	+	+	+	+	MES
22	69	M	glioblastoma	9.4	y	3.5	progression	+	-	+	-	-	-	+	-	+	+	+	-	-	PROL
25	64	M	glioblastoma	6.9	y	0.25	progression	+	-	-	-	-	-	-	-	-	-	+	+	+	MES
31	69	M	glioblastoma	1.6	n	na	na	+	un	+	-	-	-	-	-	+	+	+	-	-	PROL
32	58	M	glioblastoma	25.1	y	4	progression	+	un	-	+	+	-	-	-	-	-	+	-	-	PN
35	18	M	glioblastoma	3.5	y	0.25	progression	+	un	+	-	-	-	-	+	+	-	+	-	-	PROL
38	50	F	glioblastoma	12.5	y	1	stable	+	un	-	-	-	-	-	-	+	+	+	+	+	MES
40	58	F	glioblastoma	10.2	y	1.5	un	+	-	-	-	-	-	-	+	-	+	+	-	-	PROL
41	67	F	glioblastoma	4.2	y	un	un	+	-	+	-	+	+	-	-	-	-	+	-	-	PN
46	64	M	glioblastoma	12.7	un	na	na	+	-	-	-	-	-	-	+	+	+	+	-	-	PROL
47	74	M	glioblastoma	9.9	un	na	na	+	-	+	+	+	-	-	+	+	+	+	-	-	PROL
50	75	M	glioblastoma	16.7	y	1.5	un	+	-	-	-	-	-	-	+	-	+	+	+	+	MES
55	76	F	glioblastoma	6.7	y	un	un	+	un	+	+	-	+	-	-	-	+	+	+	-	PN
hGBM L0	43	M	glioblastoma	un	un	un	un	+	un	-	+	-	-	-	+	+	+	+	-	-	PROL
hGBM L1	45	F	glioblastoma	un	un	un	un	+	un	-	+	+	-	-	+	+	+	+	+	-	PROL
hGBM L2	30	F	glioblastoma	un	un	un	un	+	un	-	+	-	-	-	+	-	+	-	-	-	PROL
2	52	F	glioblastoma	44.9	y	10.25	improved	-	-	+	-	-	-	-	-	-	-	+	-	+	
3	56	M	glioblastoma	27	y	7.75	stable	-	-	-	-	-	-	-	-	-	-	-	-	+	MES
10	51	M	glioblastoma	40.9	y	25.75	progression	-	-	-	+	-	+	-	-	+	-	-	-	+	PN
11	46	M	glioblastoma	48.6	y	26	stable	-	+	-	-	-	+	-	-	-	-	-	-	+	MES
12	53	M	glioblastoma	24.7	y	16	progression	-	-	-	-	-	+	-	+	-	+	+	-	+	PROL
13	66	M	glioblastoma	19.2	y	7	progression	-	un	+	-	-	-	-	+	-	-	+	+	+	MES
16	70	F	glioblastoma	69.1	y	un	stable	-	-	-	+	-	-	-	-	-	-	+	+	+	MES
17	56	F	glioblastoma	6.3	y	3.75	recurrence	-	-	-	-	-	-	-	-	-	-	+	-	+	MES
18	63	M	glioblastoma	10.6	y	1.5	progression	-	un	+	+	-	-	-	-	+	-	+	-	-	PN
19	65	M	glioblastoma	13.9	y	un	un	-	-	-	-	+	-	-	-	-	+	+	-	+	MES
21	49	M	glioblastoma	14.8	y	4.5	progression	-	un	-	-	+	+	-	-	-	-	-	+	-	PN
23	69	M	glioblastoma	15.7	y	8	stable	-	-	+	-	+	-	-	-	-	+	+	-	-	PN
24	71	M	glioblastoma	38.9	y	10.5	progression	-	un	-	-	-	-	-	-	+	-	-	-	+	MES
26	60	M	glioblastoma	19.1	y	10.5	progression	-	-	-	+	-	+	-	-	-	-	+	-	-	PN
27	20	F	glioblastoma	23.6	y	2	progression	-	-	-	-	-	+	-	-	+	-	-	+	-	MES
29	66	F	glioblastoma	10	y	1.5	progression	-	+	-	-	-	+	-	-	-	-	+	-	-	PN
30	61	M	glioblastoma	4.1	n	na	na	-	un	-	-	-	-	-	-	+	+	+	-	-	PROL
33	68	F	glioblastoma	2.7	y	1.4	progression	-	-	-	+	-	-	+	-	+	-	-	-	-	PN
37	66	F	glioblastoma	14.2	y	1.25	un	-	un	-	-	-	-	-	-	-	-	-	-	-	MES
39	60	M	glioblastoma	11.6	y	1.75	un	-	-	-	+	-	-	-	-	-	-	+	-	-	PN
42	38	M	glioblastoma	15.5	y	3.5	progression	-	un	+	-	-	-	-	-	+	+	+	+	-	PROL
43	76	M	glioblastoma	6.6	y	2	progression	-	-	-	-	-	-	-	+	-	+	-	-	+	MES
48	62	M	glioblastoma	11.3	na	na	na	-	-	-	+	+	-	-	+	+	+	-	+	+	MES
49	58	M	glioblastoma	27.6	na	na	na	-	-	-	-	+	+	+	-	+	-	+	-	-	PN
51	56	M	glioblastoma	24.7	y	12	progression	-	-	-	-	-	+	+	-	+	-	-	-	+	PN
52	75	M	glioblastoma	14.8	n	na	na	-	-	-	-	+	+	+	-	-	-	-	-	-	PN
53	74	M	glioblastoma	3.1	n	na	na	-	-	-	-	-	-	-	+	-	-	+	-	-	PROL
54	73	F	glioblastoma	9.1	y	2	stable	-	-	-	-	-	-	-	-	-	-	-	-	+	MES
57	58	M	glioblastoma	4.7	un	na	na	-	un	-	-	-	-	-	-	-	-	+	+	+	MES

Key

un	unknown
na	not applicable
+	protein present
-	protein absent
PN	proneural
MES	mesenchymal
PROL	proliferative

Samples were stratified into a particular molecular subclass according to expression of most markers for this subclass.

Samples positive for more than one class were assigned to the subclass in which they showed the highest expression profile.

Table S2: Vendor information for antibodies used in this study.

Primary antibodies

**Antigen**	**Species**	**Dilution**	**Vendor**	**Catalog #**
beta-catenin	rabbit	1:200	Sigma	C2206
beta-catenin	mouse	1:200	Cell Signaling	2677
CD44	rabbit	1:1,000	Cell Signaling	3578
CD133	mouse	1:100	Miltenyi	130-092-395
CHI3L1/YKL40	goat	1:100	r&d systems	AF2599
DLL3	rabbit	1:1,000	Sigma	SAB2100594
E-cadherin	mouse	1:1,000	Invitrogen	180223
EGFR	rabbit	1:10,000	Epitomics	1902-1
EN-1	rabbit	1:1,000	Millipore	AB5732
GAPDH	mouse	1:10,000	*	
GFAP	mouse	1:500	Sigma	G6171
IDH1^{R132H}	mouse	1:20	Dianova	DIA-H09
Ki-67	mouse	1:100	Dako	M7240
MGMT	mouse	1:500	Invitrogen	35-7000
MYB	rabbit	1:10,000	Epitomics	1792-1
N-cadherin	rabbit	1:100#	Millipore	04-1126
N-cadherin - PE	mouse	1:20	BD	561554
human Nestin	mouse	1:1,000	Millipore	MAB5326
NF1	rabbit	1:100	Santa Cruz	sc-67
NOTCH1	rabbit	1:1,000	Cell Signaling	3608
OLIG2	rabbit	1:5,000	Millipore	AB9610
PCNA	mouse	1:2,000	Cell Signaling	2586
PDGF-B	rabbit	1:500	Santa Cruz	sc-7878
PTEN	rabbit	1:1,000	Epitomics	1539-1
ROBO1	rabbit	1:1,000	Invitrogen	40-5900
ROBO1	sheep	1:20	r&d systems	AF7118
SNAI1	rabbit	1:1,000	Cell Signaling	3879
SNAI2	rabbit	1:1,000	Cell Signaling	9585
SOX2	rabbit	1:1,000	Cell Signaling	3579
TOP2A	rabbit	1:1,000	Cell Signaling	4733
TWIST1	rabbit	1:1,000	Millipore	ABD29
ZEB1	rabbit	1:3,000§	Sigma	HPA027524
ZEB2	rabbit	1:3,000	Sigma	HPA003456

*Kind gift of Dr. G. Shaw, EnCor Biosciences, Gainesville, FL

Western blot: 1:50,000

§ Immunofluorescence 1:1,000

Secondary antibodies

**Antibody**	**Reporter**	**Dilution**	**Vendor**	**Catalog #**
donkey anti mouse	Alexa 555	1:500	Invitrogen	A31570
donkey anti rabbit	Alexa 488	1:500	Invitrogen	A21206
donkey anti-sheep	Alexa 568	1:500	Invitrogen	A21099
goat anti mouse	HRP	1:10,000	Bio-Rad	170-6516
goat anti rabbit	HRP	1:10,000	Bio-Rad	170-6515q
donkey anti goat	HRP	1:10,000	Jackson Immuno Research	705-035-147
